# Fruit-Surface Flavonoid Accumulation in Tomato Is Controlled by a *SlMYB12*-Regulated Transcriptional Network

**DOI:** 10.1371/journal.pgen.1000777

**Published:** 2009-12-18

**Authors:** Avital Adato, Tali Mandel, Shira Mintz-Oron, Ilya Venger, Dorit Levy, Merav Yativ, Eva Domínguez, Zhonghua Wang, Ric C. H. De Vos, Reinhard Jetter, Lukas Schreiber, Antonio Heredia, Ilana Rogachev, Asaph Aharoni

**Affiliations:** 1 Department of Plant Sciences, Weizmann Institute of Science, Rehovot, Israel; 2 Departamento de Biología Molecular y Bioquímica, Universidad de Málaga, Spain; 3 Department of Botany and Department of Chemistry, University of British Columbia, Vancouver, British Columbia, Canada; 4 Business Unit Bioscience, Plant Research International, and Centre for BioSystems Genomics, Wageningen, The Netherlands; 5 Department of Ecophysiology, Institute of Cellular and Molecular Botany, University of Bonn, Bonn, Germany; The University of North Carolina at Chapel Hill, United States of America

## Abstract

The cuticle covering plants' aerial surfaces is a unique structure that plays a key role in organ development and protection against diverse stress conditions. A detailed analysis of the tomato colorless-peel *y* mutant was carried out in the framework of studying the outer surface of reproductive organs. The *y* mutant peel lacks the yellow flavonoid pigment naringenin chalcone, which has been suggested to influence the characteristics and function of the cuticular layer. Large-scale metabolic and transcript profiling revealed broad effects on both primary and secondary metabolism, related mostly to the biosynthesis of phenylpropanoids, particularly flavonoids. These were not restricted to the fruit or to a specific stage of its development and indicated that the *y* mutant phenotype is due to a mutation in a regulatory gene. Indeed, expression analyses specified three R2R3-MYB–type transcription factors that were significantly down-regulated in the *y* mutant fruit peel. One of these, *SlMYB12*, was mapped to the genomic region on tomato chromosome 1 previously shown to harbor the *y* mutation. Identification of an additional mutant allele that co-segregates with the colorless-peel trait, specific down-regulation of *SlMYB12* and rescue of the *y* phenotype by overexpression of *SlMYB12* on the mutant background, confirmed that a lesion in this regulator underlies the *y* phenotype. Hence, this work provides novel insight to the study of fleshy fruit cuticular structure and paves the way for the elucidation of the regulatory network that controls flavonoid accumulation in tomato fruit cuticle.

## Author Summary

A major step in the evolution of land plants was the formation of a cuticular layer on their outer surfaces. Despite the cuticle's key role in organ development and in protecting against a variety of stresses, very little is known about the regulation of the metabolic pathways that generate its building blocks. Flavonoids, often embedded in the cuticle, have been suggested to impact the characteristics of this structure and to provide protection against radiation and pathogens. Flavonoids are an integral part of the human diet and are likely responsible for the observed beneficial effects of a fruit-rich diet. Here, we examine in detail the tomato colorless peel *y* mutant, which lacks the yellow flavonoid pigment naringenin chalcone, a major constituent of the fruit cuticle. Extensive transcript and metabolite profiling of this mutant revealed *SlMYB12* as a key regulator in a transcription network that controls flavonoid accumulation in tomato peel. Moreover, the change in cuticle flavonoid composition enabled us to evaluate the importance of these constituents as barriers in the cuticle layer. Finally, because most commercial cultivars in the Far East are based on the *y* genetic background, discovery of the *y* gene will contribute also to tomato breeding programs.

## Introduction

Most aerial plant surfaces are covered with a cuticle, a heterogeneous layer composed mainly of cutin and wax lipids. The cuticle is a unique surface structure that plays an important role in organ development and protection against biotic and abiotic stress conditions. Cutin is the major component of the cuticle, comprising between 40 and 80% of the cuticle's weight in various plant organs. This polyester, insoluble in organic solvents, consists of oxygenated fatty acids with a chain length of 16 or 18 carbons. Embedded in the cutin matrix are cuticular waxes, which are complex mixtures of very-long-chain fatty-acid derivatives [1]. In many species these also include triterpenoids and other secondary metabolites, such as sterols, alkaloids and phenylpropanoids, including flavonoids. For example, the flavonoid naringenin chalcone (NarCh) accumulates up to 1% dry weight of the tomato fruit cuticle: it is the yellow pigment that accumulates in wild-type (wt) fruit peel, and it is the first intermediate in the biosynthesis of flavonols. It is produced by chalcone synthase (CHS) from *p*-coumaroyl-CoA and malonyl-CoA and subsequently converted into naringenin (Nar) by chalcone isomerase (CHI) [2]. The contribution of NarCh to the characteristic features of the fruit cuticle was examined by Luque et al. [3], who suggested that it plays an important role in controlling water transport across the polymer matrix.

Apart from NarCh, various other flavonoids accumulate in tomato fruit. The flavonol rutin (quercetin-3-rutinoside), and to a lesser extent kaempferol-3-O-rutinoside and a quercetin-trisaccharide, are predominantly produced in the peel, while the fruit flesh tissues accumulate only minute amounts of flavonoids [2],[4],[5]. These biochemical data correlate with the expression of the flavonoid biosynthesis genes in tomato fruit tissues, as only low levels of flavonoid-related transcripts are detected in the flesh [4],[6]. In the peel, significant levels of the transcripts encoding CHS, flavonone 3′ hydroxylase (F3′H), and flavonol synthase (FLS) can be detected, while chalcone isomerase (CHI) mRNA levels are barely detectable [4]. Low *CHI* expression might explain the accumulation of its substrate, NarCh, in the fruit peel. In fact, transgenic tomato expressing the petunia *CHI* gene displayed increased levels of fruit peel flavonols, mainly due to the accumulation of rutin and a concomitant reduction in NarCh [2]. Thus, unlike other steps in the flavonoid pathway, only the CHI reaction seems to be blocked in tomato fruit peel, whereas most of the pathway appears to be suppressed in fruit flesh.

Verhoeyen et al. [6] reported that a reduction in NarCh in *CHI*-overexpressing tomato results in pink fruit with a dull appearance. A similar pink phenotype was obtained upon RNAi-mediated down-regulation of *CHS*, encoding the enzyme generating NarCh [7]. Total flavonoid levels, transcript levels of both *CHS1* and *CHS2*, as well as CHS enzyme activity were all significantly reduced in these latter transgenic tomato fruits. The highest RNAi-expressing lines produced extremely small and parthenocarpic fruits, and pollen-tube growth was inhibited. Scanning electron microscopy (SEM) analysis revealed that epidermal cell development is strongly disturbed in the fruit of *CHS* RNAi plants, as the typical conical cells of tomato fruit epidermis were misshapen and collapsed [7].

Recent metabolomics studies have described the range of tomato fruit phenylpropanoids, particularly flavonoids [8]–[12]. Moco et al. [11] monitored secondary metabolites during the course of tomato fruit development by several analytical tools. They revealed that flavonoids are typically present in the epidermal tissues and that various accumulation patterns during fruit development can be defined for different flavonoids. While flavonoids such as Nar and NarCh-hexose increased during fruit development, the levels of quercetin-trisaccharide decreased. Slimestad et al. [12] determined the qualitative and quantitative flavonoid compositions of various tomato cultivars. Extensive characterization revealed that the total flavonoid content of different tomato types varies from 4 to 26 mg 100 g FW^−1^, with NarCh being the predominant compound, contributing 35 to 71% of the total flavonoid content. Iijima et al. [8] performed a large-scale metabolite analysis, which identified 70 different flavonoid derivates in the flesh and peel of cv. Micro Tom (MT) at various stages of fruit development. They showed that the number of flavonoids increases during ripening and that flavonoids are more abundant in peel tissues than in the flesh. Using combined transcript and metabolite analyses, Mintz-Oron et al. [9] further demonstrated that increased activity of pathways generating cuticular lipids in tomato fruit peel precedes that of phenylpropanoid and flavonoid biosynthesis pathways.

Transcriptional regulation of the flavonoid biosynthesis pathway is achieved by the spatially and temporally coordinated expression of several transcription factors [13]–[15]. Studies in several plant species (e.g. maize, petunia, antirrhinum, Arabidopsis, tobacco, grape and apple) have revealed that members of the R2R3-MYB gene family are required for the production of anthocyanins, proanthocyanidins and flavonols [16]–[21]. Two of the best-studied examples of flavonoid-related transcription factors are the maize MYB-type *C1* and MYC-type *LC* genes. When specifically expressed in the fruit of transgenic tomato, both genes are required and sufficient for up-regulation of the flavonoid pathway in fruit flesh, which, as already mentioned, normally produces only low levels of flavonoids. This ectopic expression in tomato fruit results in a strong accumulation of flavonols (in the form of kaempferol-glycosides) and a more moderate increase in flavanones (i.e. Nar-glycosides) [4]. A recent study has shown that transgenic tomato lines overexpressing the Arabidopsis R2R3-MYB transcription factor *PAP*1, known to regulate the transcription of flavonoid-pathway genes [22], accumulate increased levels of various flavonoid derivates [8]. Luo et al. [23] studied the expression of *AtMYB12*, originally identified as a flavonol-specific transcriptional activator in Arabidopsis [18], tobacco and tomato. They showed that in tobacco, *AtMYB12* is able to induce the expression of target genes leading to the accumulation of very high flavonol levels, while in tomato, *AtMYB12* also activates the caffeoyl quinic acid biosynthetic pathway, thus confirming previous observations that transcription factors may have different specificities for target genes in different plant species [23]. In another study, Butelli et al. [24] expressed two snapdragon transcription factors in tomato fruit, the basic helix-loop-helix (bHLH) Delila (Del) and the MYB-related Rosea1 (Ros1), which are known to interact in the induction of anthocyanin biosynthesis in snapdragon flowers. Fruit of the transgenic plants accumulated high anthocyanin levels at concentrations comparable to those found in blackberries and blueberries. Expression of the two transgenes enhanced the hydrophilic antioxidant capacity of tomato fruit threefold and resulted in fruit with intense purple coloration in both peel and flesh.

In tomato, T-DNA activation-tagging experiments identified a tomato MYB-type transcriptional regulator of anthocyanin biosynthesis, named Anthocyanin1 (ANT1), which shares high homology with the petunia AN2 protein regulating late anthocyanin pathway genes [25]. Fruit of the *ant1* mutant exhibited purple spotting on the epidermis [18]. In an earlier study, Lin et al. [26] characterized the expression of 14 putative tomato MYB-type transcription factors, which showed a wide range of expression patterns, including some transcripts with marked tissue specificity.

The tomato *y* mutant was originally described in 1925 as carrying a monogenic recessive mutation leading to the formation of a colorless fruit peel, and was named “*y*” after the recessive colorless allele, in contrast to the dominant yellow “*Y*” allele [27]. In 1956, Rick and Butler [28] mapped the *y* mutation by linkage analysis to cytogenetic band 30 on the S arm of chromosome 1, the 1–30 locus. The *y*-type fruit appearance can be seen in numerous wild tomato species and cultivated varieties and is popular among commercial cultivars consumed in Asian countries (see http://phn.huji.ac.il/cgi-bin/eusolCC/index.pl).

In the framework of studying the surface of reproductive organs, we performed a detailed investigation of the *y* mutation in the tomato variety Ailsa Craig (AC). The *y* mutant fruit cuticle was thinner, exhibited lower cutin content and reduced elasticity as compared to the wt. Large-scale metabolic and transcript profiling revealed broad effects on both primary and secondary metabolism, mostly related to the biosynthesis of phenylpropanoids, particularly flavonoids. These effects were not restricted to the peel, or to a specific stage of fruit development and included alterations to the carotenoid and tocopherol branches of the isoprenoid pathway as well. One of three down-regulated R2R3-MYB transcription factors, *SlMYB12*, was mapped to the genomic region on chromosome 1 that had been previously shown to harbor the *y* mutation [28]. Further confirmation that *SlMYB12* is the regulator underlying the *y* phenotype was obtained through (i) the identification of a second *SlMYB12* allele that co-segregates with the colorless-peel trait in an unrelated population, (ii) its down-regulation by a synthetic microRNA approach and (iii) phenotype complementation upon overexpression of *SlMYB12* on the *y* genetic background. In this study, we therefore describe the identification of a transcription factor that regulates the biosynthesis of a major cuticular component in tomato fruit, and provide new insights into the formation of soft fruit cuticular structure.

## Results

### The *y* Mutant Phenotype

**Figure 1:**
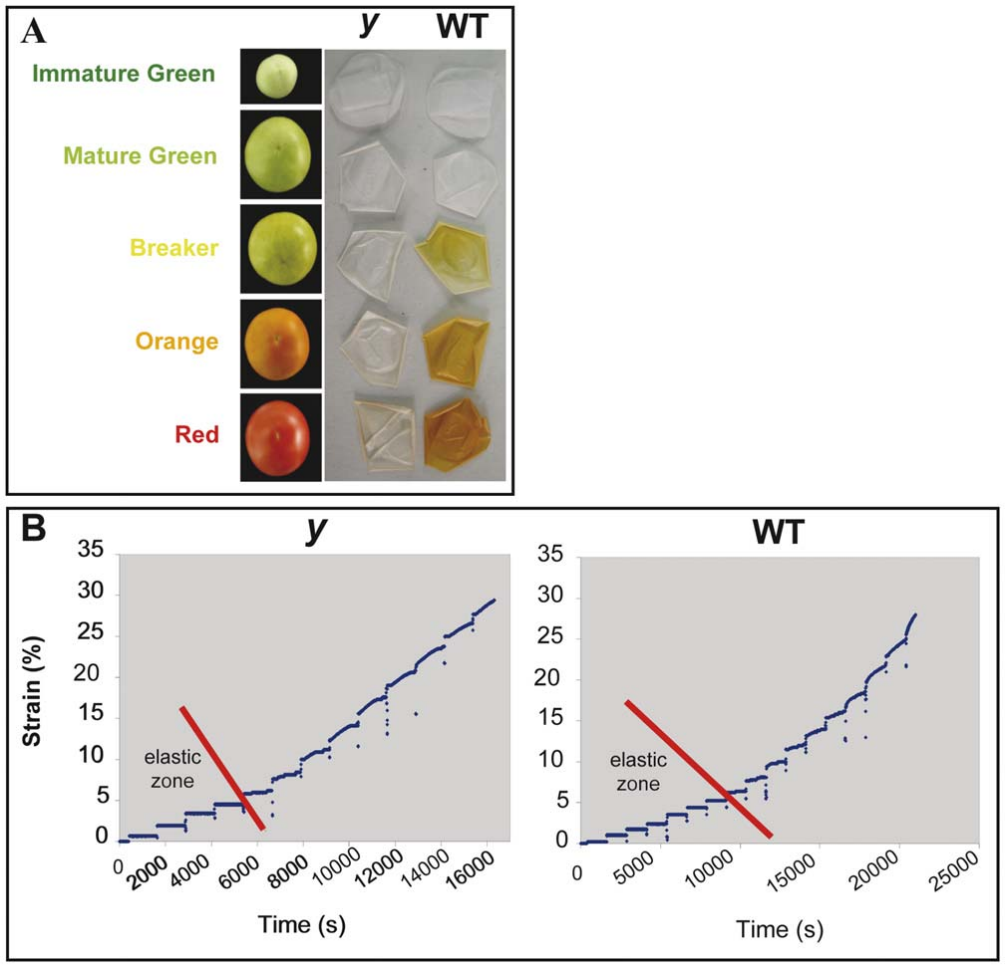
The *y* mutant phenotype and its cuticular properties. (A) Enzymatically isolated cuticles (n = 20) of wt and *y* mutant at five stages of fruit development. (B) Biomechanical tests of isolated fruit cuticles (n = 7) at the red stage of development reveal significant differences in the elastic phase between wt and *y* mutant cuticles.

The tomato *y* mutant, examined here as part of our effort to characterize the unique features of reproductive organ surfaces, was described long ago as carrying a monogenic recessive colorless fruit peel mutation [27],[29]. In contrast to the wt, *y* mutant fruit do not accumulate the yellow pigment that typically suffuses throughout the cell walls of wt fruit epidermis (Figure 1A). The *y* fruit exhibits a pink and less glossy appearance at the late orange and red stages of fruit development. Unlike the fruit of the *CHS*-silenced plants described by Schijlen et al. [7], *y* fruits are not parthenocarpic and an analysis of their surface by electron microscopy did not reveal any significant difference from the wt fruit. Apart from their fruit, *y* plants do not exhibit a visible phenotype in any other plant organs.

### Cuticular Properties Are Altered in the *y* Mutant Fruit

As previously described [9], manually dissected tomato peel samples may contain the cuticle, the epidermis and several cell layers beneath it. To investigate the cuticular properties, peel tissues manually dissected from *y* and wt fruits were treated with pectinase and cellulase. Thickness measurements of cross sections from the isolated cuticle fragments revealed that the wt cuticle is significantly thicker (8.01±0.08 µm) than the *y* cuticle (6.22±0.12 µm; n = 30).

To examine the distribution of flavonoids in the fruit cuticle, isolated cuticle fragments from ripe fruits were examined through a fluorescein isothiocyanate (FITC) filter. Whereas faint fluorescence could be detected in the wt cuticle, no fluorescence was observed in the *y* mutant cuticle (Figure S1A), suggesting that most of the fluorescence observed in the wt cuticle is due to the presence of flavonoids. Further evaluation of flavonoids present in the isolated cuticles was performed by diphenylboric acid-2-aminoethyl ester (DPBA) staining. Like the FITC filter, DPBA staining illustrated the absence of flavonoids in the *y* cuticle (Figure S1B).

Luque et al. [3] suggested that the flavonoids Nar and NarCh play an important role in the control of water transport across the cuticle. We therefore compared the water transpiration of isolated cuticular fragments (Figure S1C). This analysis revealed a trend of decrease in transpiration (i.e. water permeance) of wt cuticles during fruit development that was not detected in *y* cuticles (Figure S1C). Moreover, no significant difference in the rate of water loss was found between post-harvest whole *y* and wt fruit which were left to dry out at under constant temperature and humidity (Figure S1D). An unexpected finding was revealed during these water-loss tests: while wt fruit shrank and their peel became wrinkled, most of the *y* fruit did not (Figure S1E). Nevertheless, by manual inspection it was evident that the inner tissues of the *y* fruit were collapsing as the fruit became soft, while the shrunken wt fruit stayed relatively firm. This phenotype was clearly manifested by placing half of a dissected fruit in water: the wt tomato sank as one intact piece to the bottom of the beaker, while the peel of the *y* fruit separated from its degrading inner tissues (Figure S1E). This observation suggested that the cell walls from the inner tissues of *y* fruit undergo earlier disassembly than those of wt fruit.

Biomechanical tests of isolated cuticles revealed no significant differences between the wt and *y* mutant in elastic modulus (441.72±34.27 and 455.69±41.58 MPa, respectively) or in breaking force (55.06±10.26 and 53.83±15.08 MPa, respectively). However, a significant difference between wt and *y* cuticles was detected in the length of their elastic phase, which reached 0.686 N (Newton) and 0.392 N in isolated cuticle fragments of wt and *y* fruit, respectively (Figure 1B).

### The *y* Mutant Phenotype Is Not a Result of Mutations in the Genes Encoding CHS1 and 2

A pair of *CHS* genes (*SlCHS1* and *SlCHS2*) are preferentially expressed in tomato fruit peel and their expression pattern during fruit development is correlated with the expression of other flavonoid pathway structural genes and the accumulation of NarCH [7],[9]. Quantitative Real-Time (RT) PCR analysis of *SlCHS1* and *SlCHS2* in both wt and *y* mutant AC fruits revealed down-regulation of their transcripts in the *y* mutant at all three tested stages of fruit development (Breaker, orange and red) (Figure S2A). Overexpression of *CHS1* in tomato resulted in a *y*-like phenotype which was sectorial in some cases (Figure S2B and S2C), most probably due to *CHS* co-suppression. These results suggested that a mutation in the *CHS* gene(s) might be the cause for the *y* mutant phenotype. To examine this possibility, both genes were isolated and sequenced from wt and *y* mutant plants. Apart from several cases of intronic single nucleotide polymorphisms (SNPs), no difference that might alter their translated protein was detected in the sequences of *SlCHS1* and *SlCHS2* of the *y* mutant. Furthermore, overexpressing *SlCHS1* in the *y* background did not complement the mutant phenotype (data not shown). Thus, although their expression is down-regulated in the *y* fruit, *SlCHS1* or *SlCHS2* are not the genes underlying the *y* mutant phenotype.

### Microarray Analysis Reveals that Transcriptional Changes in the *y* Mutant Are Not Confined to the Peel: They Also Occur in the Flesh Tissue

Transcriptome analysis was carried out to compare gene expression in *y* and wt fruit (in both peel and flesh tissues) at three developmental stages (breaker, orange and red). A total of 406 non-redundant transcripts exhibited twofold or more increased or decreased expression in the *y* mutant vs. wt peel or flesh at at least one of the three tested stages of fruit development (Table S1). Most of these transcripts (353 out of 406) differed at only one developmental stage, 56 transcripts differed at two developmental stages, and 16 differed at all three developmental stages (Table S1). Sixty of the 406 transcripts differed in both flesh and peel tissues. The differences in expression of 17 selected transcripts (putatively identified as tomato *PDH*, *PAL*, *C3H*, *4CL*, *CHS1, CHS2, CCR, CHI, F3H, FLS, RT, C3H, CCR, THM27, MYB4-like, SlNCED* and *SlCRTR-B2*) were confirmed by means of quantitative RT-PCR analyses.

**Figure 2:**
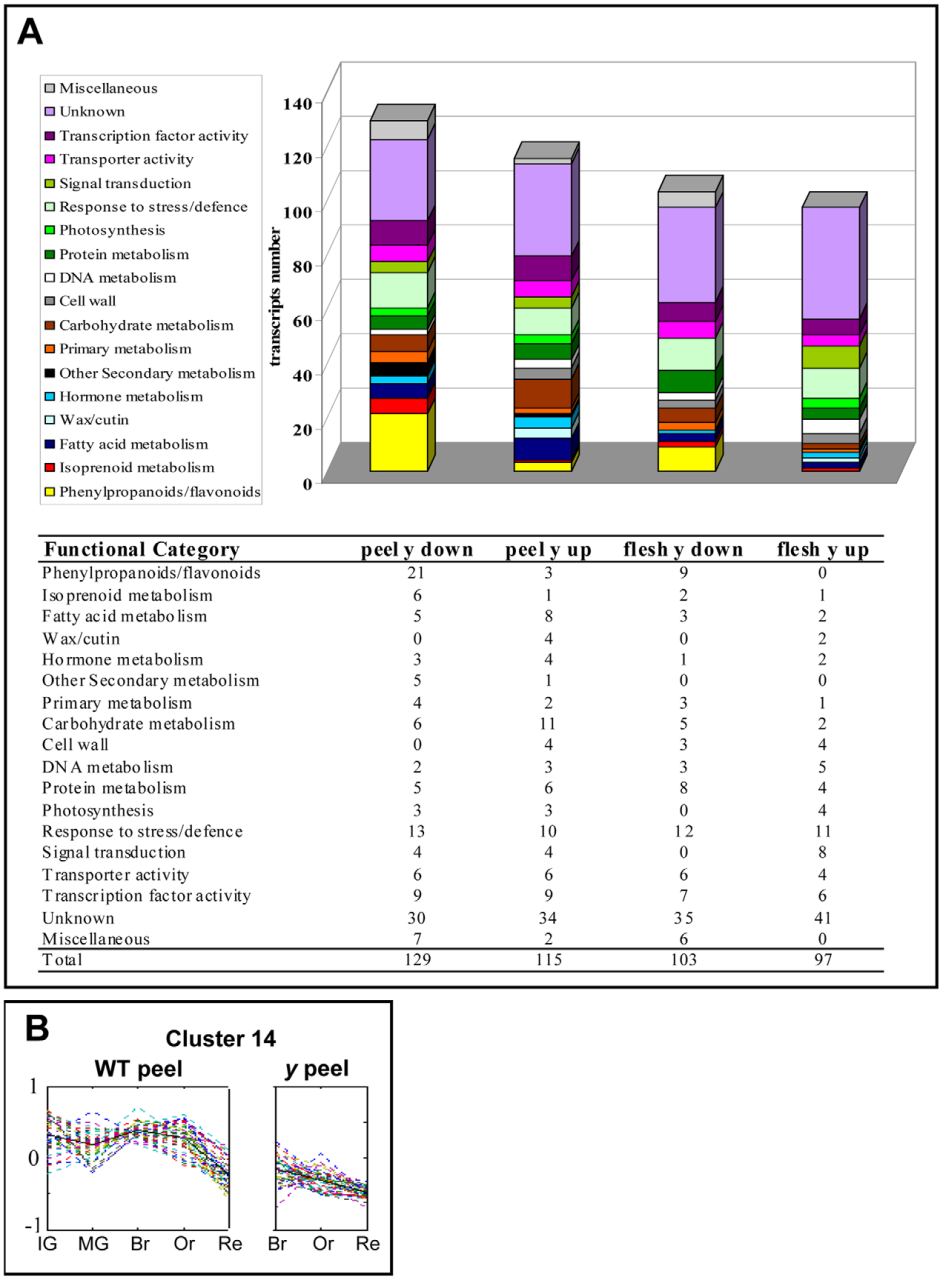
Gene-expression alterations in the *y* mutant fruit as revealed by array analysis. (A) Functional category distribution among differentially expressed wt and *y* mutant transcripts at the three latest stages of fruit development. (B) The expression profile (obtained by array analysis) of genes belonging to cluster 14 (total 38 members) in the peel tissue of *y* mutant and wt fruit. In the *y* mutant, array analysis was carried out on three of the five stages of fruit development examined in the wt fruit.

The differential transcripts were assigned to putative functional categories based on sequence similarities to studied homologues/orthologues from other species (Figure 2A). The most represented functional category included 21 phenylpropanoid/flavonoid-related transcripts that were down-regulated in the *y* mutant fruit peel; nine of these transcripts were down-regulated in the flesh as well. Six transcripts putatively associated with isoprenoid metabolism were also down-regulated in the *y* peel. On the other hand, two groups of putative carbohydrate and fatty acid metabolism-related transcripts (11 and 8, respectively) were up-regulated in the *y* peel. Various transcription factors were up- or down-regulated in *y* mutant tissues (Table S1), among them two members of the R2R3-MYB family, *SlTHM27* and *SlMYB4-like* (TC174616 and TC184379, respectively), i.e. tomato homologues of the *AtMYB4* flavonoid-related transcription factor, which were down-regulated in both peel and flesh tissues of the *y* mutant.

To study the expression patterns of genes differing between *y* and wt during fruit development, all 406 differentially expressed transcripts were clustered. Forty expression-profile clusters were created, 20 for flesh and 20 for peel (Figure S3). Cluster #14, for example, is composed of 38 transcripts that exhibited lower expression in the *y* peel (Figure 2B). These down-regulated peel genes included 15 transcripts putatively related to phenylpropanoid/flavonoid metabolism, including two transcription factors (*SlTHM27* and *SlMYB4like*), six transcripts associated with response to stress and defense, two transcripts related to fatty-acid metabolism and 15 transcripts from other categories. Eleven transcripts belonging to this cluster (including eight phenylpropanoid/flavonoid-related ones) were down-regulated in the *y* peel at all three developmental stages (Table 1).

**Table 1:** Genes down/up-regulated in peel or flesh tissues of the *y* mutant at the three tested stages of tomato fruit development (breaker, orange, red).

Identifier	Gene annotation^a^	Pathway	Cluster^b^
**Down-regulated in ** ***y*** ** fruit peel at the three tested developmental stages**			
BI209975^c^	Lipase	Fatty acid metabolism	14
TC178705	Chalcone isomerase (CHI)	Phenylpropanoids/flavonoids	14
TC180957	Flavanone 3-hydroxylase (F3H)	Phenylpropanoids/flavonoids	14
TC176277	Flavonoid 3-glucosyl transferase (3GT)	Phenylpropanoids/flavonoids	14
TC170658	Chalcone synthase (CHS1)	Phenylpropanoids/flavonoids	14
TC170429	Phenylalanine ammonia-lyase (PAL)	Phenylpropanoids/flavonoids	14
TC179039	Rhamnosyltransferase (RT)	Phenylpropanoids/flavonoids	14
TC180112	Cinnamoyl CoA reductase-like (CCR)	Phenylpropanoids/flavonoids	14
TC176549	Flavonoid 3-glucosyl transferase (3GT)	Phenylpropanoids/flavonoids	14
TC186636	C-4 sterol methyl oxidase (SMO)	Isoprenoid	14
TC178916	Putative glycine-rich RNA binding protein	Unknown	17
TC187382	Unknown	Unknown	14
**Up-regulated in ** ***y*** ** fruit peel at the three tested developmental stages**			
AW039066	Lipase (EXL1)	fatty acid metabolism	5
TC177136	Annexin	Unknown	20
TC173084	Unknown	Unknown	20
**Down-regulated in ** ***y*** ** fruit flesh at the three tested developmental stages**			
TC171069	Unknown	Unknown	11
**Up-regulated in ** ***y*** ** fruit flesh at the three tested developmental stages**			
TC177136	Annexin	Unknown	14

a Putative annotation of transcripts and pathways are based on the closest known homologues/orthologues from other species.

b Peel/flesh expression profile clusters to which the gene belongs (see Figure S3 for clusters).

c GB accessions are given when no TC index (TIGR identifier) is available.

### Both Primary and Secondary Metabolism Are Affected in the Developing *y* Mutant Fruit

To examine the effect of the *y* lesion on fruit metabolism, we carried out a comprehensive Metabolomics analyses of *y* and wt tissues during five stages of fruit development (immature green, mature green, breaker, orange and red). Various analytical methods were employed including: ultra-performance liquid chromatography coupled to a quadrupole time-of-flight mass spectrometry (UPLC-QTOF-MS) for the detection of semi-polar components (mainly secondary metabolites), gas chromatography-MS (GC-MS) analysis for the identification of polar compounds (mainly primary metabolites), GC with flame ionization detector (GC-FID) for the profiling of waxes in isolated fruit cuticles, and HPLC coupled to UV and fluorescence detectors for the analysis of lipid-soluble isoprenoids.

**Figure 3:**
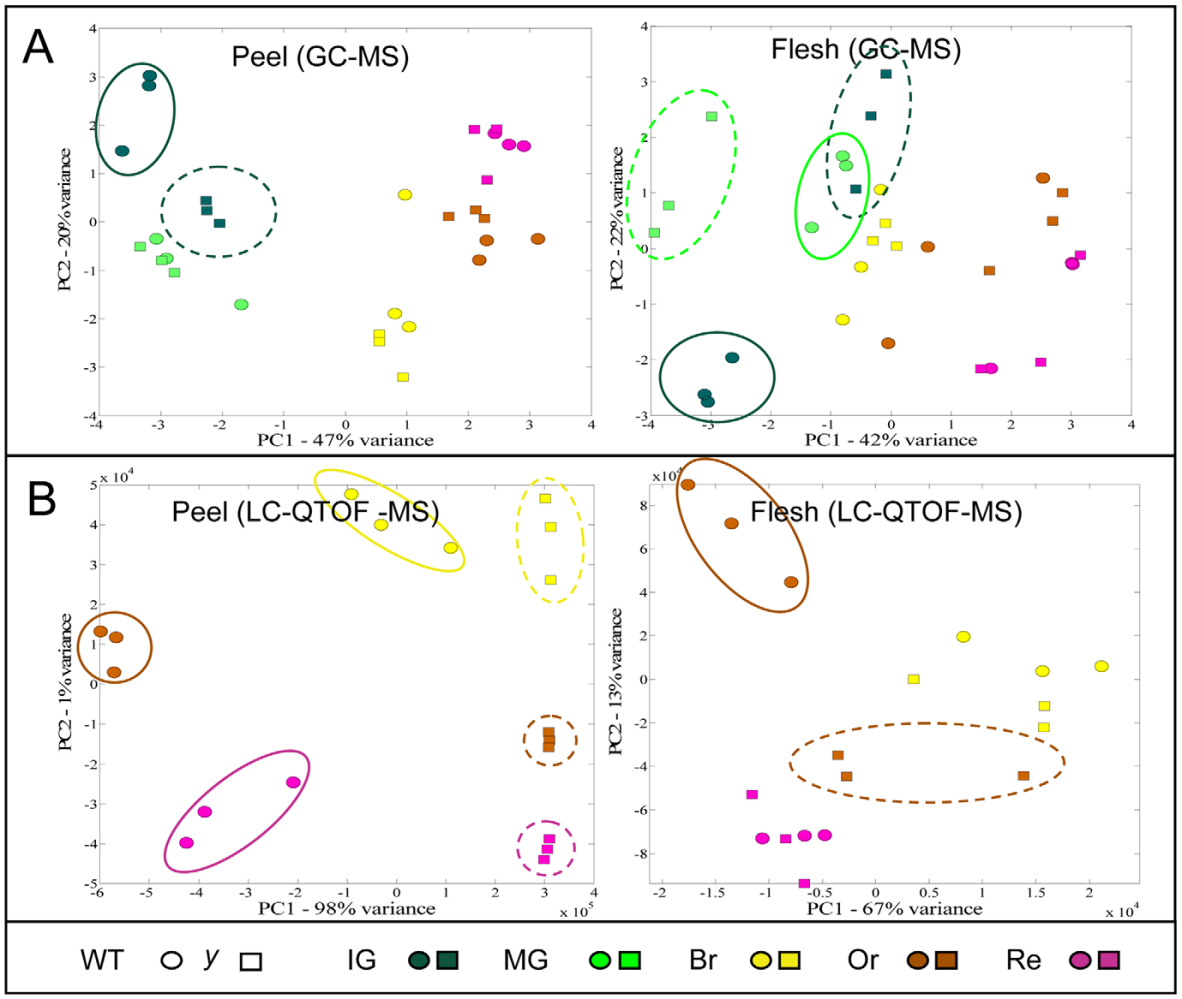
Differences between metabolic profiles of wt and *y* mutant peel and flesh tissues detected by principal component analysis (PCA) of GC-MS and LC-QTOF-MS data sets. (A) PCA of metabolic profiles obtained by GC-MS analysis, with samples of wt and *y* peel and flesh tissues at five stages of fruit development (n = 3). (B) PCA of metabolic profiles obtained by UPLC-QTOF-MS analysis, with samples of wt and *y* peel and flesh tissues in the last three stages of fruit development (n = 3). Distinct metabolic profiles that correspond to particular stages of *y* and wt fruit development are encircled in (A,B).

To obtain a general view of the differences in metabolite profiles between *y* and wt fruit tissues, a principal component analysis (PCA) was conducted with the metabolite GC-MS and LC-MS data sets (Figure 3). In the GC-MS set, PCA could distinguish between the metabolite profiles of *y* and wt fruit at early stages of development, i.e. at the immature green stage in the peel and at the immature and mature green stages in the flesh (Figure 3A). PCA of the UPLC-QTOF-MS data set (negative ESI mode), derived from the peel tissue at five stages of development, could distinguish between the *y* and wt metabolite profiles in the last three stages of fruit development. Such differences were not evident in the flesh tissue samples, where all five tested stages of fruit development were analyzed together (Figure S4). However, when PCA was carried out on a data set that excluded the two early stages (immature and mature green), a clear distinction was demonstrated between the metabolic profiles of *y* and wt peels in the three late stages and in flesh samples at the orange stage (Figure 3B).

Thus, at early stages of fruit development, the differences between *y* and wt metabolic profiles are mainly due to changes in the levels of polar (mostly primary) metabolites detected by GC-MS analysis. Differences between *y* and wt fruit in secondary metabolites (mostly detected by UPLC-QTOF-MS) appear at later stages of fruit development, predominantly in the peel tissue.

### The Levels of Primary Metabolites, Particularly Amino and Organic Acids, Are Reduced in *y* Fruits at Early Stages of Development

**Figure 4:**
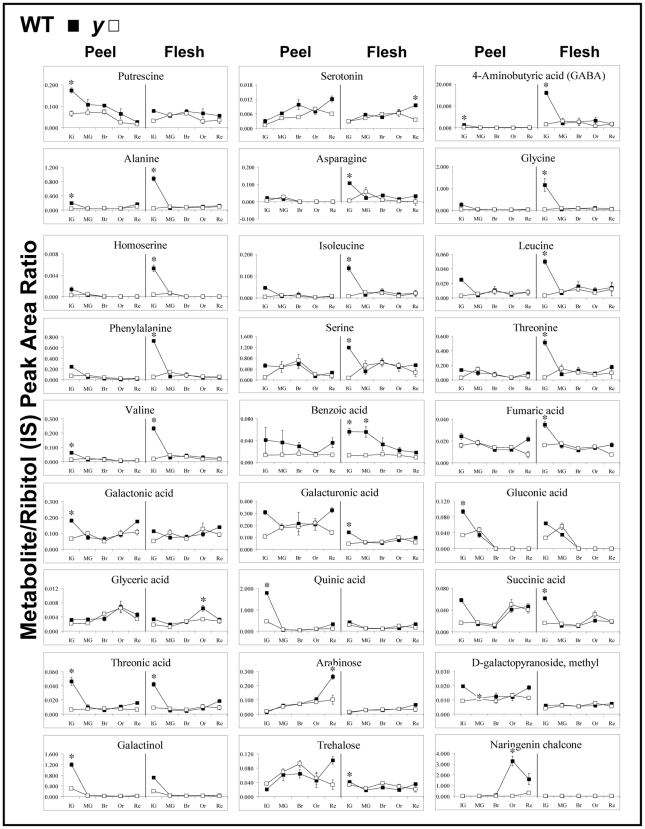
GC-MS analyses detected 27 out of 56 assigned polar metabolites (Mintz-Oron et al. [6]) as having significantly different levels between wt and *y* mutant peel and/or flesh at at least one tested stage of fruit development. Indicated by asterisks are significant differences as analyzed by two-way ANOVA and post-hoc analyses, see Materials and Methods (n = 3 for each sample). Y axis indicates metabolites' relative quantification by normalization of their response values to the Ribitol internal standard (IS) (see also Mintz-Oron et al. [6]).

Out of the 56 metabolites that could be monitored by the GC-MS technology, most of which were primary metabolites, 27 (including two amines, 11 amino acids, nine organic acids, four sugars and NarCh) differed significantly between *y* and wt tissues at at least one stage of fruit development (all exhibited reduced levels in *y* fruit tissues; Figure 4). Most of the differential metabolites (23 out of 27) showed reduced levels at the immature green stage of *y* fruit. The other four differential metabolites - NarCh, arabinose, glyceric acid and the amine serotonin - significantly differed at the orange and red stages. Four differential metabolites - 4-aminobutyric acid (GABA), alanine, valine and threonic acid - were significantly different between *y* and wt fruit in both peel and flesh tissues, 15 were significantly different only in the flesh tissue and eight were different only in the peel tissue. Interestingly, two phenylpropanoid precursors, phenylalanine and benzoic acid, significantly differed between *y* and wt in immature green fruit, but only in the flesh tissue. To summarize, levels of various primary metabolites, particularly amino acids and organic acids, were reduced in early *y* fruit development, mostly in the flesh tissue.

### The Extensive Alterations to Gene Expression and Metabolism Associated with the Phenylpropanoid and Flavonoid Pathways Are Not Restricted to the *y* Mutant Peel

**Figure 5:**
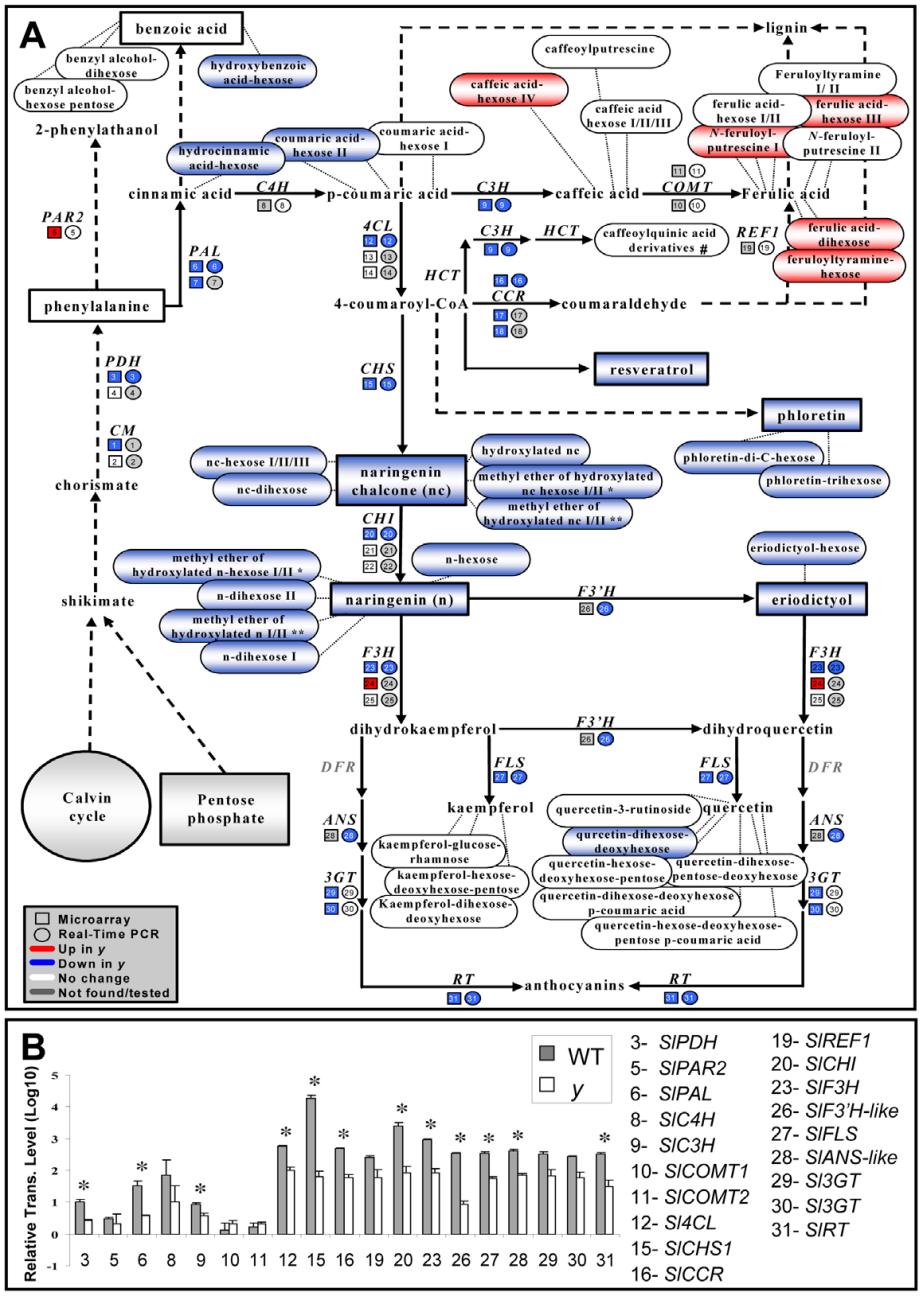
Alterations in metabolite and gene-expression levels in the phenylpropanoid pathway as detected in the *y* mutant fruit peel tissue. (A) Changes in gene expression (detected using array and/or real-time PCR analyses) and metabolite levels (detected by UPLC-QTOF-MS and GC-MS analyses) in tomato fruit peel. Red and blue colors represent up- and down-regulation, respectively. (B) Real-time PCR relative expression analyses of selected transcripts from the phenylpropanoid pathway in wt and *y* mutant tomato peels at the breaker stage of fruit development. Indicated by asterisks are significant differences analyzed by Student's t-test (n = 3; *P*<0.05; bars indicate standard errors). Gene identifiers and RT–PCR primers are listed in Table S3. #, caffeoylquinic acid derivatives that include: 4-caffeoylquinic acid, 5-caffeoylquinic acid, dicaffeoylquinic acid I/II/III, tricaffeoylquinic acid and 3-dicaffeoylquinic acid, detected but not altered in both peel and flesh of the *y* mutant. Metabolites marked by * or ** may be NarCh or Nar derivatives.

UPLC-QTOF-MS metabolite analysis resulted in the assignment of 71 putative, mostly secondary metabolites in developing tomato fruit tissues (Table S2). According to a two-way ANOVA test, 29 of these metabolites significantly differed between *y* and wt fruit tissues (Table 2). All differential metabolites were products of the phenylpropanoid and flavonoid pathways (Figure 5A and Figure S5A).

**Table 2:** UPLC-QTOF-MS-detected metabolites that are differentially produced between *y* mutant and wt fruit peel tissues.

Peak No.^a^	Putative metabolite	Molecular formula	Differential stage
**Metabolites down-regulated in the ** ***y*** ** mutant peel**			
59	Hydrocinnamic acid-hexose	C_15_H_20_O_8_	Br, Or, Re^b^
39	Coumaric acid-hexose II	C_15_H_18_O_8_	Or
42	*trans*-Resveratrol (S)	C_14_H_12_O_3_	Br, Or, Re
23	NarCh^c^ (S)^d^	C_15_H_12_O_5_	Br, Or, Re
25	NarCh-hexose I	C_21_H_22_O_10_	Or, Re
26	NarCh-hexose II	C_21_H_22_O_10_	Or, Re
27	NarCh-hexose III	C_21_H_22_O_10_	Or, Re
24	NarCh-dihexose	C_27_H_32_O_15_	Or, Re
35	Hydroxylated NC	C_15_H_12_O_6_	Br, Or, Re
22	Nar^e^ (S)	C_15_H_12_O_5_	Or, Re
28	Nar-hexose	C_21_H_22_O_10_	Or
29	Nar-dihexose I	C_27_H_32_O_15_	Or, Re
30	Nar-dihexose II	C_27_H_32_O_15_	Or, Re
36	Hydroxylated Nar (Eriodictyol) (S)	C_15_H_12_O_6_	Or, Re
37	Hydroxylated Nar-hexose (eriodictyol-hexose)	C_21_H_22_O_11_	Or
31	Methyl ether of hydroxylated N or Methyl ether of hydroxylated NarCh I	C_16_H_14_O_6_	Or, Re
34	Methyl ether of hydroxylated N or Methyl ether of hydroxylated NarCh II	C_16_H_14_O_6_	Br, Or, Re
32	Methyl ether of hydroxylated N-hexose, or Methyl ether of hydroxylated NarCh-hexose I	C_22_H_24_O_11_	Or, Re
33	Methyl ether of hydroxylated N-hexose, or Methyl ether of hydroxylated NarCh-hexose II	C_22_H_24_O_11_	Or, Re
64	Phloretin (S)	C_15_H_14_O_5_	Br, Or, Re
65	Phloretin-di-C-hexose	C_27_H_34_O_15_	Or, Re
66	Phloretin-trihexose	C_33_H_44_O_20_	Or, Re
16	Quercetin-dihexose-deoxyhexose	C_33_H_40_O_21_	Or
41	Hydroxybenzoic acid-hexose	C_13_H_16_O_8_	Or
**Metabolites up-regulated in the ** ***y*** ** mutant peel**			
63	Caffeic acid-hexose IV	C_15_H_18_O_9_	Re
60	Ferulic acid-dihexose	C_22_H_30_O_14_	Or
61	Ferulic acid-hexose III	C_16_H_20_O_9_	Or
57	*N*-Feruloylputrescine I	C_14_H_20_N_2_O_3_	Or
69	Feruloyltyramine-hexose	C_24_H_29_NO_9_	Or

a The peak number here corresponds to the numbers given for all metabolites detected by the UPLC-QTOF-MS analysis in this study (see details in Table S2).

b Br, Or and Re are for breaker, orange and red stages of fruit development, respectively.

c NarCh - naringenin chalcone.

d (S) - compound was identified by comparison of its retention time and mass spectrum with those of the authentic standard.

e Nar-naringenin.

The two large groups of flavonoids detected in the peel tissue were NarCh and Nar derivatives, and quercetin derivatives. Apart from a single derivative, all members of the NarCh/Nar group were down-regulated in the *y* mutant peel, while the levels of most quercetin derivatives were not altered (Figure 5A). Other flavonoids identified in *y* and wt peels were eriodictyol and one of its derivatives, and two kaempferol derivatives, with only eriodictyol and its derivative being down-regulated in the *y* mutant peel. Additional phenylpropanoids and flavonoids were also detected in the peel, including benzoic acid and two of its derivatives, three coumaric acid derivatives, six caffeic acid derivatives, seven ferulic acid derivatives, phloretin and two of its derivatives, and trans-resveratrol. Of these, only one benzoic acid derivative, one coumaric acid derivative, phloretin and its two derivatives, and the trans-resveratrol were down-regulated in the *y* mutant fruit peel. Secondary metabolites that showed enhanced levels in *y* fruit were four ferulic acid derivatives and a single caffeic acid derivative, which are all part of the phenylpropanoid pathway branch associated with lignin metabolism.

As indicated above, microarray gene-expression analysis revealed the down-regulation of 21 transcripts putatively associated with the phenylpropanoid/flavonoid pathway in the *y* mutant peel (Figure 2A). These included early shikimate pathway- and general phenylpropanoid-related transcripts (*SlPDH* and *SlCM*, and *Sl4CL* and *SlCCR*, respectively), transcripts corresponding to the flavonoid pathway and associated with NarCh and Nar biosynthesis (e.g. *SlCHS*, *SlCHI* and *SlF3H*), and *SlFLS* transcripts that putatively catalyze the formation of flavonols. The down-regulation of most of these genes was corroborated by the results of RT-PCR analysis, which included five additional putative phenylpropanoid/flavonoid-related genes (*SlANS*, *SlF3′H*, two *SlCOMT* genes and *SlREF1*) not present on the array (Figure 5B, Table S3). Expression levels of the two additional genes, *SlCOMT* and *SlREF1*, which are putative structural genes in the lignin metabolism branch and related to the biosynthesis of ferulic acid derivatives, did not differ between the *y* mutant and wt peels. According to the array results, three putative phenylpropanoid/flavonoid-related genes (*SlPAR*, *SlF3H* and a tomato acyltransferase) appeared to be up-regulated in at least one of the tested stages of fruit development. However, in the case of the *SlPAR* gene, RT-PCR analysis did not confirm the microarray results, and in the case of *SlF3H*, a different putative *SlF3H* was found to be down-regulated in the *y* mutant peel by both the array and RT-PCR analyses. Overall, gene expression according to both microarray and RT-PCR analyses further corroborated the wide alterations exhibited by the phenylpropanoid/flavonoid pathway in the *y* mutant peel tissue.

While the levels of phenylpropanoid/flavonoid metabolites were also altered in the *y* mutant flesh, the changes were much less pronounced than in the peel tissue (Table 3, Figure S5A). This finding is in accordance with many previous studies showing lower activity of the phenylpropanoid/flavonoid pathway in tomato flesh [2], [4], [5], [9]–[11]. NarCh, Nar, phloretin and their derivatives were also found to be down-regulated in the *y* mutant flesh tissue as compared to that of the wt. Worth noting are phenylalanine and benzoic acid from the upper part of the phenylpropanoid pathway, which showed significant down-regulation only in the flesh of the *y* mutant. Feruloyltyramine hexose, *N*-feruloylputrescine and caffeoylputrescine in the lignin-related branch of the phenylpropanoid pathway were up-regulated in both flesh and peel of the *y* mutant (Figure S5A). Although overall, the expression of all structural genes from the phenylpropanoid/flavonoid pathway was lower in the flesh than in the peel (of both *y* and wt fruit), their down-regulation in *y* (relative to wt expression levels) was also evident in the flesh (Figure S5B). Thus, extensive alterations in the phenylpropanoid/flavonoid pathway were also detected in the flesh of the *y* mutant.

**Table 3:** UPLC-QTOF-MS detected metabolites that are differentially produced between *y* mutant and wt fruit flesh tissues.

Peak No.^a^	Putative metabolite	Molecular formula	Differential stage
**Metabolites down-regulated in the ** ***y*** ** mutant flesh**			
23	NarCh^b^ (S)^c^	C_15_H_12_O_5_	Br, Or, Re^d^
26	NarCh-hexose II	C_21_H_22_O_10_	Or, Re
22	Nar^e^ (S)	C_15_H_12_O_5_	Or
29	Nar-dihexose I	C_27_H_32_O_15_	Or, Re
65	Phloretin-di-C-hexose	C_27_H_34_O_15_	Or, Re
66	Phloretin-trihexose	C_33_H_44_O_20_	Or, Re
**Metabolites up-regulated in the y mutant flesh**			
58	*N*-Feruloylputrescine II	C_14_H_20_N_2_O_3_	Re
69	Feruloyltyramine-hexose	C_24_H_29_NO_9_	Or
70	Caffeoylputrescine	C_13_H_18_N_2_O_3_	Re

a The peak number here corresponds to the numbers given for all metabolites detected by the UPLC-QTOF-MS analysis in this study (see details in Table S2).

b NC - naringenin chalcone.

c (S) - compound was identified by comparison of its retention time and mass spectrum with those of the authentic standard.

d Br, Or and Re are for breaker, orange and red stages of fruit development, respectively.

e N - naringenin.

Examining the expression of six structural and three regulatory phenylpropanoid/flavonoid-associated genes in leaves of *y* and wt plants revealed a clear trend of down-regulated expression for all of the tested genes in fully expanded *y* leaves (Figure S6A). However, the expression of these genes did not differ between *y* and the wt in young leaves (Figure S6B). In addition, PCA analysis of metabolite data sets obtained by UPLC-QTOF-MS profiling of roots derived from *y* and wt seedlings clearly distinguished between the profiles of the two genotypes (Figure S6C). Thus, the effects of the lesion underlying the *y* mutant phenotype are not restricted to fruit tissues.

### Besides the Lack of Naringenin Chalcone, Wax-Constituent Composition and Cutin Content Are Also Altered in *y* Mutant Fruit Cuticle

**Figure 6:**
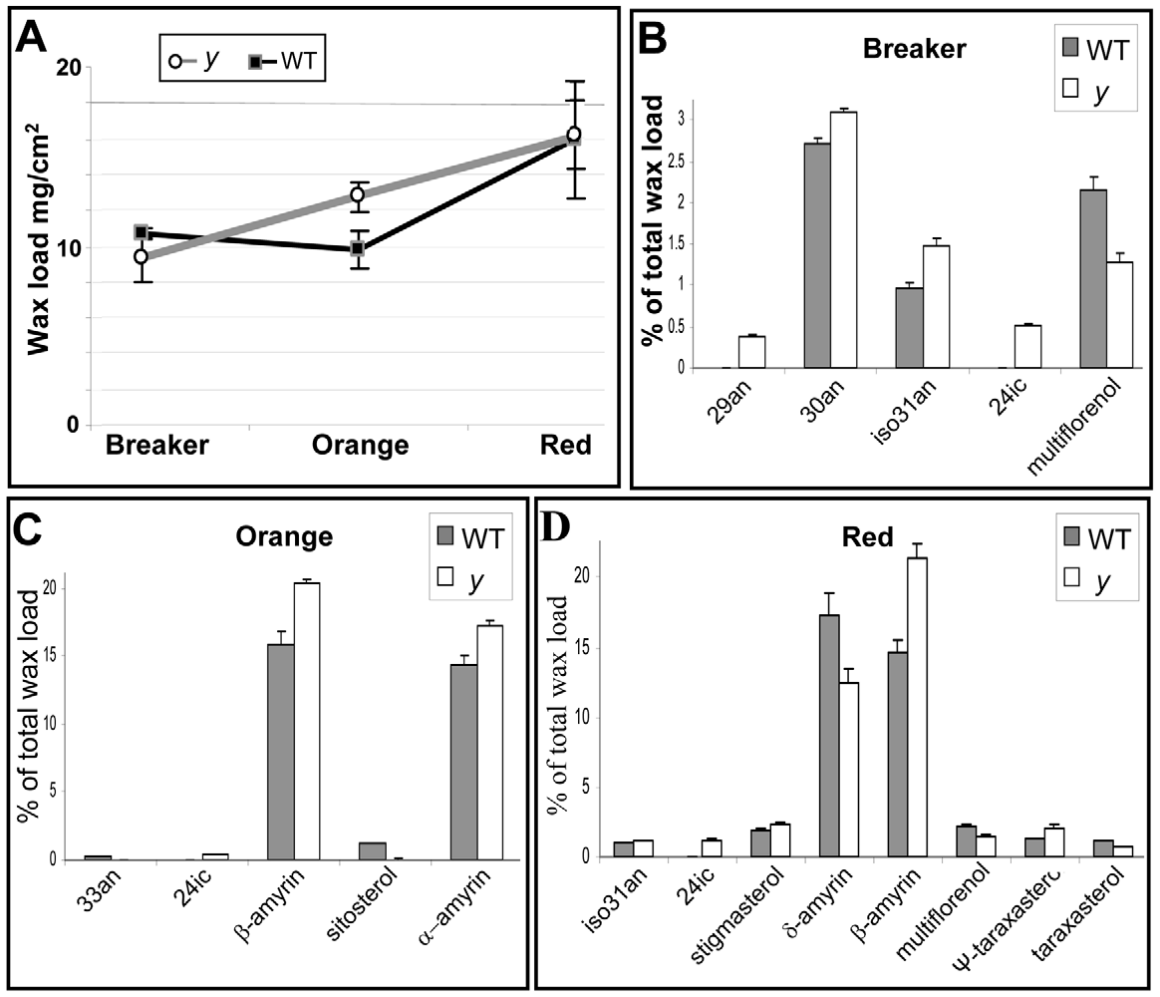
Composition of the *y* mutant and wt fruit cuticular waxes was tested at three stages of fruit development, analyzed by GC-MS and GC-FID (n = 5; *P*<0.05; bars indicate standard errors). (A) Total cuticular wax load expressed as mg/cm^2^. (B) Amounts (%) relative to total wax coverage of individual compounds that were significantly altered at the breaker stage of fruit development, (C) at the orange stage of fruit development, and (D) at the ripe red stage of fruit development. 29an, C_29_ alkane; 30an, C_30_ alkane; iso31an, C_31_ iso alkane; 24ic, tetracosanoic acid.

The macroscopic appearance of *y* fruit at late stages of development, the lack of NarCh and the changes in elasticity, suggested that the *y* cuticle is altered in its chemical composition. A targeted chemical analysis of isolated cuticles from both *y* and wt fruit at three stages of development (breaker, orange and red fruit) was therefore performed. Cuticular waxes were extracted from isolated cuticles and the remaining cutin matrix was then depolymerized by BF_3_/methanol for cutin analysis. A total of 20 compounds were identified in the wax mixtures, including two steroids (stigmasterol, *β*-sitosterol) and seven pentacyclic triterpenoids (taraxerol, *δ*-amyrin, *β*-amyrin, *α*-amyrin, multiflorenol, Ψ-taraxasterol and taraxasterol), and a homologous series of branched and unbranched alkanes (C_29_–C_33_), C_24_ fatty acid (tetracosanoic acid) and C_32_ primary alcohol (dotriacontanol) (Figure S4). An altered pattern of total wax accumulation was observed in the *y* mutant cuticles due to a significant difference at the orange stage (Figure 6A). While in wt cuticles waxes accumulate mainly between the orange and red stages of fruit development, wax accumulation in *y* cuticles is constant from the breaker to the red stages. Figure 6B–6D presents the individual compounds that were significantly altered in *y* fruit during development. While minor differences were detected in several alkanes and sterols, the major alterations were in the levels of terpenoids (*α*-, *β*-, and *δ*-amyrins as well as multiflorenol) (Figures 6B–6D and Figure S7). The similarity in total wax coverage between the two genotypes at the red stage (Figure 6A) could be explained by the opposite trends in alteration of *β*-, and *δ*-amyrins.

In addition, two other components of the isolated cuticles, namely cutin and polysaccharides, were examined in cuticles from ripe red fruit. While only a slight difference was detected in the polysaccharide levels between wt and *y* mutant cuticles (499.4±13.2 and 533.8±8.7 µg cm^−2^, respectively; n = 10), cutin content was significantly higher in the wt fruit cuticle (1397.6±36.8 and 1170.3±19.1 µg cm^−2^, respectively; n = 10). However, no significant difference was detected in the composition of cutin monomers (data not shown).

### Transcripts and Metabolites Associated with the Isoprenoid Pathway Are Also Down-Regulated in the *y* Mutant Fruit

**Figure 7:**
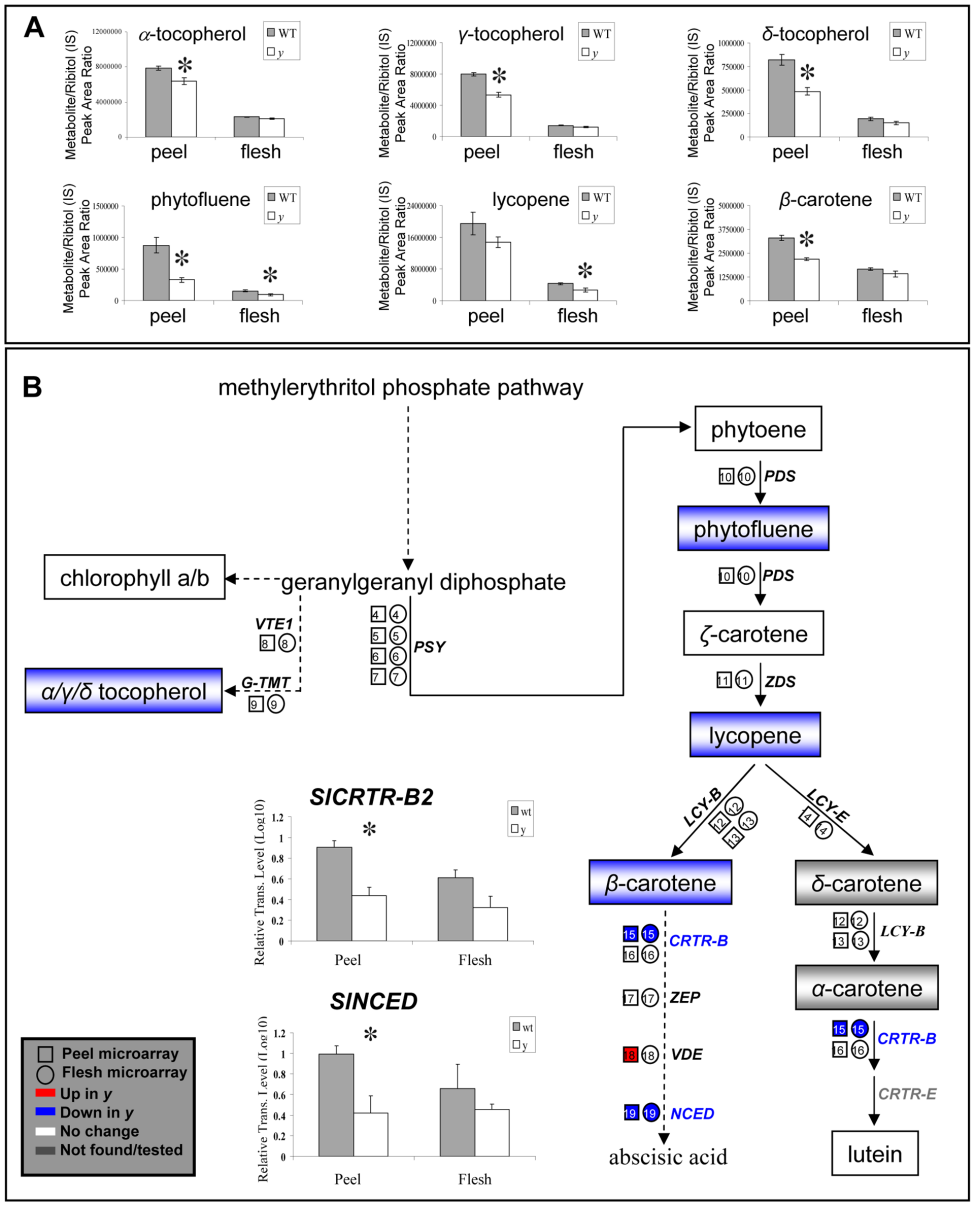
Changes in metabolite and gene-expression levels in the isoprenoid pathway detected in *y* mutant fruit tissues. (A) HPLC-PDA analysis detected six isoprenoids that differ significantly between the wt and *y* mutant fruit tissues at the orange stage of fruit development. Indicated by asterisks are significant differences analyzed by Student's t-test (n = 3; *P*<0.05; bars indicate standard errors). (B) Changes in isoprenoid-related gene expression (detected by array analysis) and metabolite levels (detected by HPLC-PDA) in tomato fruit peel and flesh tissues. Red and blue colors represent up- and down-regulation, respectively. Significant down-regulation of *SlCRTR-B2* and *SlNCED* transcript levels in the *y* mutant peel was confirmed by RT–PCR analyses (Student's t-test, n = 3; *P*<0.05; bars indicate standard errors). Primers, transcript identifiers/accessions and expression data are listed in Table S4.

As already noted, the microarray analysis revealed six transcripts putatively related to the isoprenoid pathway that are down-regulated in the *y* mutant fruit peel (Figure 2, Table S1, and Table S4), two of which (putative carotenoid-related *SlNCED* and *SlCRTR-B2*) were also down-regulated in the flesh. Significant down-regulation of these two transcripts in the *y* fruit peel was confirmed by RT-PCR analysis (Figure 7). On the other hand, one putative carotenoid-related transcript (*SlVDE*) was up-regulated in the *y* peel and one putative isoprenoid-related transcript (putative *SlIPPI*) was up-regulated in the *y* flesh. HPLC analysis of lipid-soluble isoprenoids revealed that 6 out of the 11 analyzed metabolites (Table S5) are significantly down-regulated in the *y* fruit tissues, namely tocopherols *α*, *γ* and *δ* and *β*-carotene (lower in the peel), all-trans lycopene (lower in the flesh) and phytofluene (lower in both peel and flesh; Figure 7A). A summary of all modifications in the isoprenoid pathway is presented in Figure 7B.

### *SlMYB12*, One of the Three Flavonoid-Related R2R3-MYB Transcription Factors Down-Regulated in the *y* Mutant Peel, Is Mapped to the *y* Locus on Tomato Chromosome 1

The broad effects on gene expression and metabolism in *y* suggested that a gene upstream of *SlCHS*, possibly a regulatory factor, is responsible for the *y* mutant phenotype. Microarray analysis revealed down-regulated expression of two members of the R2R3-MYB transcription factor family, *SlTHM27* and *SlMYB4-like* (TC174616 and TC184379, respectively) in *y* fruit at the breaker and orange stages. However, sequencing of the coding regions of both transcripts from *y* and wt fruit cDNA samples revealed SNPs that are not expected to alter the function of the putatively translated proteins. Furthermore, these two transcription factors were mapped to chromosomes 10 and 6 and not to the previously known *y* mutation locus on chromosome 1 [28].

**Figure 8:**
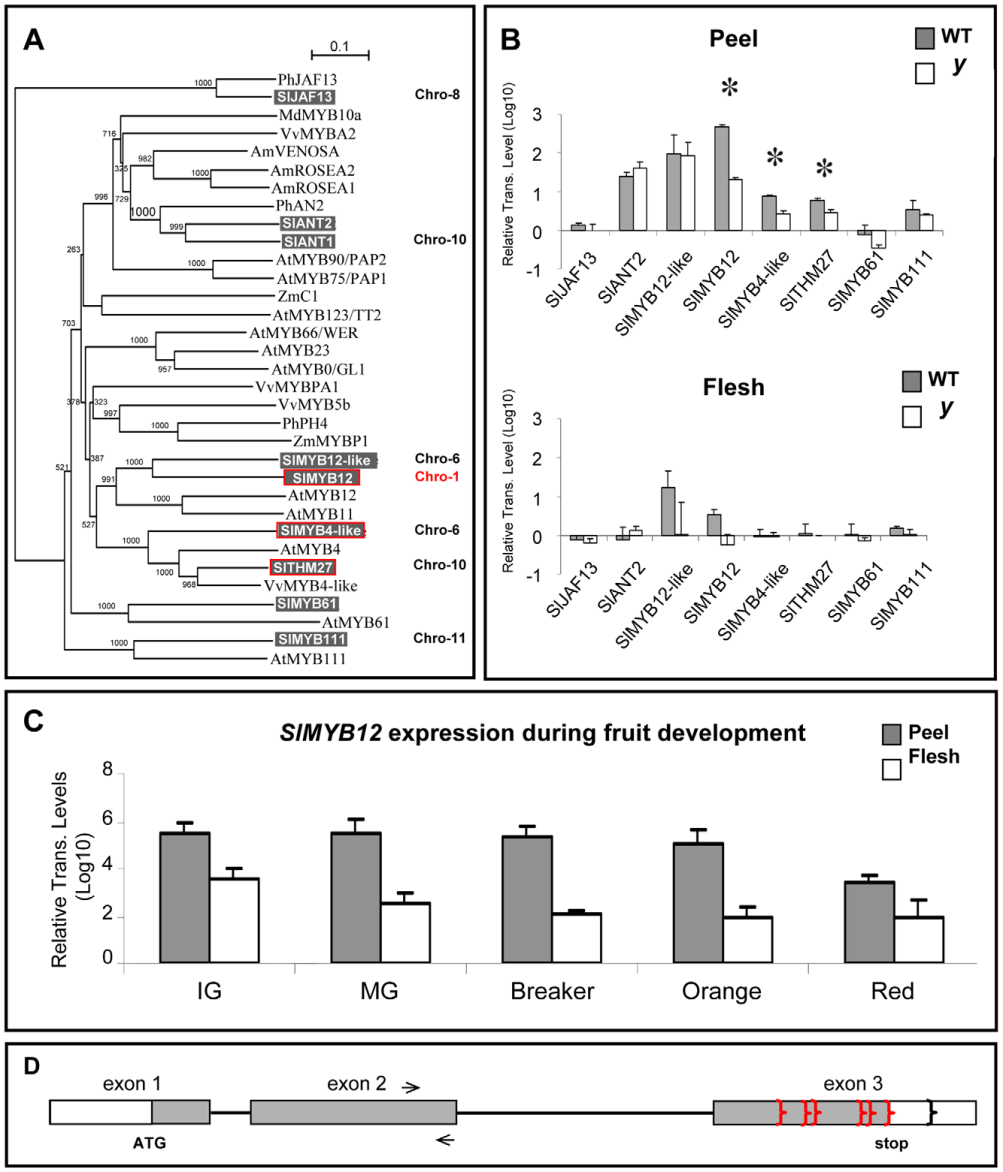
Phylogeny and expression analyses of putative phenylpropanoid/flavonoid-related transcription factor genes. (A) Phylogenetic analysis of the putative tomato regulators reported in our study and known phenylpropanoid/flavonoid-related transcription factors from other species. The ClustalX and NJplot softwares were used to compute the tree and its significance (bootstrap) values. (B) RT–PCR relative expression analyses of selected tomato transcription factors putatively related to the regulation of the phenylpropanoid/flavonoid pathway in wt and *y* mutant tissues at the breaker stage of fruit development. Indicated by asterisks are significant differences analyzed by Student's t-test (n = 3; P<0.05; bars indicate standard errors). Gene identifiers and primers are listed in Table S3. (C) Real Time RT–PCR relative expression analysis of *SlMYB12* in wt fruit tissues through five developmental stages reveals a peel-associated expression pattern. IG, Immature Green; MG, Mature Green; Br, Breaker; Or, Orange; Re, Red, stages of fruit development (n = 3; P<0.05; bars indicate standard errors). (D) Structure of the *SlMYB12* gene. Gray regions represent coding sequence and white represents UTRs. Arrows indicate the position of RT–PCR primers, red brackets indicate the positions of premature polyadenylation sites (in the *y-1* mutant allele), and the black bracket indicates the position of the alternative wt polyadenylation site.

To determine the regulatory factor responsible for the *y* mutant phenotype, we identified and reconstructed seven additional putative tomato transcription factors that are orthologues/homologues of known flavonoid-related regulators from other species (six R2R3-MYB family members and one bHLH). Phylogenetic analysis performed with the predicted protein sequences of these tomato regulators and sequences of known flavonoid-related transcription factors from other species (Figure 8A) revealed three paralogous pairs: SlMYB12 and SlMYB12-like, SlMYB4-like and SlTHM27 and SlANT1 and SlANT2, which are putative orthologues/homologues of the Arabidopsis MYB12 and MYB4 and the petunia AN2 transcription factors, respectively. Two additional tomato R2R3-MYB genes (*SlMYB111* and *SlMYB61*) are orthologues of the Arabidopsis *MYB111* and *MYB61*, and the bHLH transcription factor (*SlJAF13*) is an orthologue of the petunia *JAF13*. Sequencing of transcripts corresponding to the seven additional putative regulators from both *y* and wt did not yield any sequence lesions that would be likely to alter the function of their putatively encoded proteins. Expression analysis revealed that only one of these additional regulators (*SlMYB12*) exhibits altered expression levels in the *y* mutant fruit tissues (Figure 8B). Chromosome locations were assigned to five of these seven candidate regulators. *SlMYB12, SlMYB12-like*, *SlMYB111* and *SlJAF13* were mapped to chromosomes 1, 6, 11 and 8, respectively, while *SlANT1* has been recently mapped to tomato chromosome 10 [30]. Partial overlaps between the *pennellii* chromosome segments inserted into the interspecific introgression lines (ILs) used for the gene mapping enabled localization of *SlMYB12* to an interval between cM 17 and 41 on chromosome 1, which contains the previously defined *y* mutation locus, 1–30 (Figure S8). Analysis of *SlMYB12* expression during the five stages of fruit development demonstrated peel-associated expression that was maximal at the immature green stage and declined as the fruit developed towards the red ripe stage (Figure 8C).

Thus, no tomato regulatory gene was found to harbor a mutation likely to alter the function of its predicted protein. Three out of the nine studied transcription factors (*SlTHM27*, *SlMYB4-like* and *SlMYB12*) were significantly down-regulated in the *y* mutant fruit peel, but only one of these, the peel-associated *SlMYB12*, mapped to a genomic region on chromosome 1, previously reported to harbor the mutation underlying the *y* mutant phenotype [28].

### Another Allele Co-Segregating with the Colorless-Peel Trait in an Unrelated Tomato Population Supports the Notion that *SlMYB12* Is Responsible for the *y* Mutant Phenotype

The reconstructed structure of the *SlMYB12* gene consists of 2200 bp and includes two introns (Figure 8D). Random amplification of cDNA ends (RACE) analysis revealed two alternative polyadenylated versions at the 3′ UTR of the wt *SlMYB12* transcript at nucleotid positions 73 (S version) and 204 (L version) downstream of the stop codon (Figure 8D). Sequencing of the genomic *SlMYB12* from the AC cultivar and the *y* mutant yielded several SNPs, but none of these were specific to the *y* genotype. So far, more than 1.3 kb of the *SlMYB12* upstream region have been reconstructed and sequenced. However, no differences between *y* and wt sequences have been detected in this sequenced promoter region.

In addition, we sequenced the *SlMYB12* gene from some other colorless-peel tomato lines derived from different origins. Several ILs, generated by an interspecific cross between a tomato elite processing inbred line (*Solanum lycopersicum* E6203) and the wild species *Solanum neorickii* (previously known as *L. parviflorum*) (LA2133)[31], were found to carry the same combination of sequence alterations in the introns and exons of their *MYB12* gene. These alterations, which also included some SNPs and a 3-bp deletion, are expected to cause five missense changes (K227M, R237E, V245A, N256S and T331A) and one amino acid deletion (N315del) in the corresponding protein (Figure S9A). Prediction programs (PSIPRED http://bioinf.cs.ucl.ac.uk/psipred/; JPRED http://www.compbio.dundee.ac.uk/~www-jpred/index.html) determined that these amino acid changes are likely to decrease the protein's stability, especially the T331A missense change which is expected to disturb the formation of a helix structure at the C terminus. Furthermore, 3′ RACE analysis performed on *SlMYB12* transcripts from these colorless-peel lines revealed that the multiple sequence changes introduce a new signal(s) for premature polyadenylation of the *SlMYB12* transcripts, at amino acids 237, 264, 273, 320 and 327 within the coding sequence of exon 3 or just in the stop codon (Figure 8D and Figure S9B). The integrated sequence changes comprising this putative *y* allele (termed *y-1*) were found to co-segregate with the colorless-peel phenotype among more than 100 lines of the examined *Solanum neorickii* IL population and were not detected in the *SlMYB12* genes isolated from the M82, MT, AC and E6203 cultivars.

### An Artificial MicroRNA Targeting *SlMYB12* Induces a *y*-Like Phenotype in Transgenic Plants, While Constitutive Expression of the Gene Leads to Phenotype Complementation

**Figure 9:**
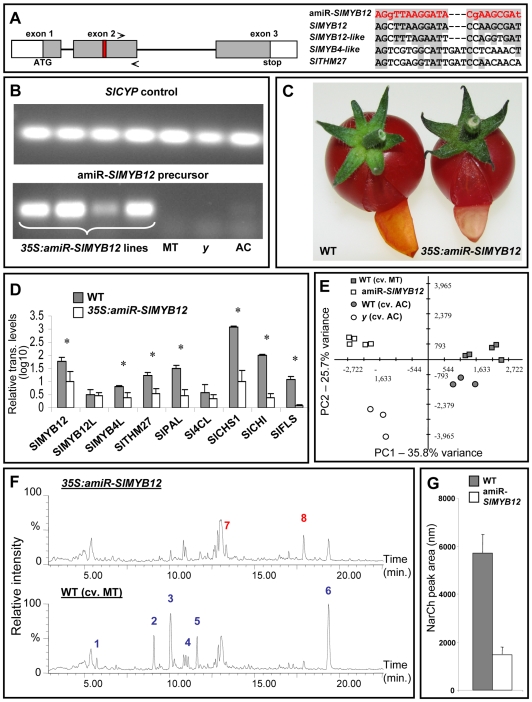
The transgenic amiR-*SlMYB12* lines exhibit a *y*-like phenotype. (A) Red box indicates the location of the amiR-*SlMYB12* target sequence on the *SlMYB12* gene, arrows indicate the position of RT–PCR primers, and the sequence alignment on the right demonstrates the specificity of this artificial microRNA. (B) Expression of the amiR-*SlMYB12* precursor in samples extracted from leaves of *35S:amiR-SlMYB12*-transgenic lines and non-transgenic controls. (C) Fruit of *35S:amiR-SlMYB12*-transgenic lines display colorless peel. (D) RT–PCR relative expression analysis of phenylpropanoid/flavonoid-related regulators and structural genes in fruit peel of wt and *35S:amiR-SlMYB12-*transgenic lines. Indicated by asterisks are significantly reduced levels analyzed by Student's t-test (n = 3; *P*<0.05; bars indicate standard errors). (E) PCA of metabolic profiles obtained by UPLC-QTOF-MS analysis carried out on peel samples of wt cv. AC and cv. MT, *y* mutant and an amiR-*SlMYB12*-transgenic line at the red stage of fruit development. Analysis was performed with the TMEV program using normalized and log-transformed data. (F) Total ion chromatograms (TICs) of wt (cv. MT) and *35S:amiR-SlMYB12* peels at the red stage of fruit development, acquired in the negative mode using UPLC-QTOF-MS (in relative intensity, 100% corresponds to 6.14×10^4^ counts). The putative identity of the differential compounds is: 1 - quercetin-dihexose-deoxyhexose, 2 - quercetin-hexose-deoxyhexose-pentose, 3 - quercetin-rutinoside (rutin), 4 - phloretin-di-C-hexose, 5 - kaempferol-glucose-rhamnose, 6 - naringenin chalcone, 7 - dicaffeoylquinic acid III, 8 - tricaffeoylquinic acid. Red and blue numbers indicate metabolites that showed elevated or reduced levels in the transgene samples in comparison to those of their corresponding wt, respectively. (G) Relative levels of NarCh in cv. MT and *35S*:*amiR-SlMYB12*, expressed as chromatographic peak areas, calculated for m/z 271.06 Da (n = 5).

To confirm the assumption that *SlMYB12* is the regulator underlying the *y* phenotype, an artificial microRNA that specifically targets *SlMYB12* (amiR-*SlMYB12*) was designed and expressed in tomato (cv. MT) under the control of the constitutive CaMV 35S promoter (Figure 9A and 9B). Fruit derived from six transgenic lines exhibited the typical *y* mutant colorless-peel phenotype (Figure 9C). RT-PCR analysis revealed significant down-regulation of *SlMYB12* as well as of the two additional transcription factors, *SlTHM27* and *SlMYB4-like*, which were also down-regulated in the *y* mutant. Significantly reduced expression levels were also detected for phenylpropanoid/flavonoid-related structural genes, including *SlPAL*, *SlCHS*, *SlCHI*, and *SlFLS* (Figure 9D). Expression of *SlMYB12-like*, the closest paralogue of *SlMYB12*, was not altered in the amiR-*SlMYB12*-expressing plants.

PCA of the metabolic profiling data (LC-MS) obtained from peel samples of ripe fruit of the amiR-*SlMYB12* and its wt (cv. MT), as well as of the *y* mutant and its wt (cv. AC), clearly distinguished between the profiles of amiR-*SlMYB12* and wt cv. MT (Figure 9E). The metabolite profiles of wt peels from cv. MT and cv. AC differed as well, but were much closer to each other than to the *y* mutant or amiR-*SlMYB12* profiles. Significant down-regulation in the levels of several flavonoids, including NarCh, phloretin di-hexoside and several flavonol conjugates, as well as up-regulation in the levels of a few caffeic acid derivatives, were also detected in the amiR-*SlMYB12*-expressing plants (Figure 9F and 9G and Figure S10).

**Figure 10:**
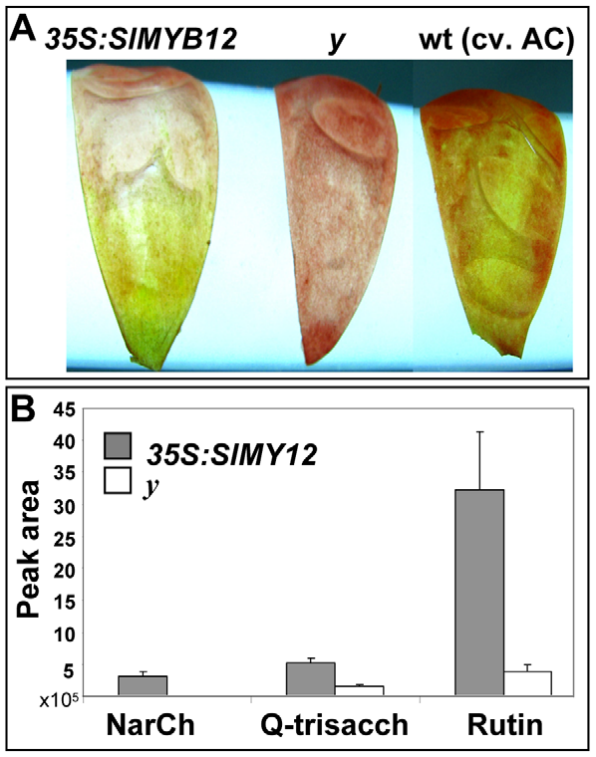
Sectorial phenotype complementation in a transgenic *y* line constitutively expressing the *SlMYB12* gene under the 35S CaMV promoter (*35S:SlMYB12*). (A) Peels of red fruit from the *35S:SlMYB12* line on a *y* background, the *y* mutant and wt cv. AC. (B) UPLC-PDA analysis of red fruit peels reveals significantly different levels of flavanoids between regions of phenotype complementation in peels of the *35S:SlMYB12* line and those of the *y* mutant (n = 3; *P*<0.01; bars represent standard error). The UPLC instrument used in this analysis is equipped with an Acquity 2996 PDA detector. Sample preparation and LC conditions were as described for the UPLC-QTOF-MS analysis. Compounds peak areas were determined by Empower 2 software (Waters) at 370 nm for naringenin chalcone (NarCh) and at 256 nm for quercetin-hexose-deoxyhexose-pentose (Q-trisacch) and quercetin-rutinoside (rutin).

Furthermore, expression of *SlMYB12* (driven by the 35S CaMV promoter) on the *y* mutant background resulted in fruit displaying phenotype complementation, as several large yellow sectors were evident in their peels (Figure 10A). UPLC-PDA (photodiode array) analysis of red fruit peels revealed the accumulation of NarCh in the yellow peel sectors derived from plants overexpressing *SlMYB12*. Moreover, additional flavonoids, such as quercetin-hexose-deoxyhexose-pentose and rutin, also accumulated in these plants (Figure 10B).

## Discussion

The tomato colorless peel *y* mutant was used in this study as a tool to investigate fruit-surface biology. The *y* fruit exhibits a severe reduction in NarCh, the yellow flavonoid pigment that typically accumulates to up to 1% of the cuticle mass. Detailed characterization of *y* fruit tissues, as well as of some other plant parts, revealed extensive alterations in the transcripts and metabolites associated with the phenylpropanoid/flavonoid pathway, which were not restricted to the fruit peel. Being part of the cuticular structure, the loss of NarCh and possibly other cuticle-related flavonoids affected fruit cuticle composition, thickness and elasticity. Changes were also detected in transcripts and metabolites related to the carotenoid and tocopherol branches of the isoprenoid pathway. The discovery that down-regulation of *SlMYB12* underlies the *y* mutant phenotype uncovered a putative transcriptional network that controls flavonoid accumulation in the tomato fruit surface.

### The *y* Mutant Tissues Display Down-Regulation of Both Transcripts and Metabolites Associated with the Phenylpropanoid Pathway

In a previous study, we showed that a dramatic accumulation of flavonoids, one of the dominant classes of secondary metabolites in tomato fruit peel, precedes the formation of cuticular lipids [9], initiating close to the breaker stage of fruit development. The major differences in secondary metabolism between *y* and wt fruit tissues were observed at the orange stage and were mostly related to alterations in the phenylpropanoid/flavonoid pathway. A correlation between down-regulated transcripts and metabolite levels in this pathway was revealed in most of the biosynthetic-pathway steps, including the *SlPAL* and *Sl4CL* genes and their related metabolites in the upper part of the pathway, as well as *SlCHS*, *SlCHI* and NarCh/Nar and their derivatives. The reduction in the levels of glycosylated metabolites along the pathway, such as coumaric-acid-hexose II and several NarCh- and/or Nar-hexoses, might be due to reduced expression of glycosyl transferases such as *Sl3GT* and *SlRT*. However, enzyme-activity assays are required to determine the substrate specificity of these typically promiscuous enzymes.

The correlation between down-regulated transcripts and metabolites appeared to be weaker in the pathway branch leading to the biosynthesis of flavonols, in which significantly reduced expression levels of *SlFLS* in the *y* peel led to the reduction of only one single flavonol species (quercetin-dihexose-deoxyhexose), while the levels of all other detected flavonol derivatives did not differ from those in the wt. One possible reason for this might be related to the relatively early accumulation pattern of flavonols in cv. AC as compared to other flavonoids [9], so that flavonols might be less affected by the *y* mutation, which is most apparent in the breaker and orange stages of development. Unlike the *y* mutant (in the cv. AC background), amiR-*SlMYB12* transgenic plants (in the cv. MT background) exhibited significant down-regulation of several flavonols (e.g. quercetin-hexose-deoxyhexose-pentose and rutin) in the fruit peel. This could be related to differences in flavonol accumulation between the two genetic backgrounds.

In contrast to the overall down-regulation in gene expression and metabolite levels associated with the phenylpropanoid/flavonoid pathway, levels of some metabolites related to this pathway's lignin side branch were up-regulated in the *y* mutant, mostly ferulic acid derivatives. Albeit weaker, this effect was also significant in the *y* flesh tissue. However, this increase in metabolite levels, which was evident in both tissue types, was not accompanied by alterations in gene expression. We therefore suggest that this effect results from a shift in the metabolic flux through the phenylpropanoid pathway rather than being a direct outcome of changes in the transcriptional regulation of this branch.

Interestingly, levels of the core phenylpropanoid precursor, the amino acid phenylalanine, were found to be significantly down-regulated only in the *y* flesh and not in its peel tissue. This result might be explained by more extensive use of this phenylpropanoid amino acid precursor, which prevents its accumulation in the peel tissue. Alternatively, it might indicate that the major synthesis of the phenylalanine precursor takes places in the flesh, from where it is translocated to peripheral epidermal layers. Such tissue translocation of precursors and intermediates has been previously suggested for phenylpropanoids, terpenoids and alkaloids, as well their biosynthetic intermediates, which are known to be synthesized in parenchymatous cells before their accumulation and storage in other tissues [32].

### Alteration in Cuticle Flavonoid Content Is Associated with Modifications in Other Cuticle Components

Metabolites present in the cuticle, such as the amyrins and NarCh, serve as structural elements for this layer. Their proportion of the total cuticular waxes, physical characteristics and spatial arrangement/assembly within the cuticle may affect properties of the cuticular barrier (e.g. elastic modulus and water loss). In addition to the significant change in NarCh levels, differences were also observed between *y* and wt cuticles in the levels of other cuticular constituents, predominantly the cyclic triterpenoid *β*-amyrin and to a lesser extent *α*- and *δ*-amyrins, waxes (mainly alkanes) and total cutin. The flavonoids Nar and NarCh have been suggested to play an important role in the control of water transport across the cuticle [3]. However, despite the chemical changes observed in its cuticle, no significant alteration was detected in the water permeance of the *y* mutant (Figure S2A). Similarly, characterization of the Delayed Fruit Deterioration mutant (DFD)[33], which also lacks NarCh in its fruit peel, showed minimal transpirational water loss. Those authors suggested that increased amounts of wax and cutin, coupled to the absence of NarCh in DFD, resulted in denser packing of the cutin matrix and in increased cross-linking of the cutin polymer at the ripe red stage of fruit development [34]. In a different study, Leide et al. [35], suggested that the increase in water permeance of the *lecer6* tomato mutant was related to a decrease in the proportion of *n*-alkanes and a concurrent increase in levels of cyclic triterpenoids in the *lecer6* cuticle. As mentioned above and presented in Figure 6, variable alterations of *y* cuticle triterpenoid levels (increased levels of *α*- and *β*-amyrin and decreased levels of *δ*-amyrin) and slightly increased levels of a few alkanes did not yield any significant alteration in the mutant's water permeance.

### The Predominant Down-Regulation of Phenylpropanoids in the *y* Mutant Is Coupled to a Reduction in Carotenoid and Tocopherol Levels

Apart from the changes in phenylpropanoid/flavonoid composition and levels, the *y* mutant displays down-regulation in the levels of metabolites and transcripts related to the isoprenoid pathway. The metabolic alterations include the tocopherol and carotenoid branches of the pathway, although these changes are not correlated with detectable changes in gene expression. Upstream steps in the isoprenoid pathway, such as those generating tocopherols, have been shown to react to changes in the levels of carotenoids. For instance, transgenic plants overexpressing phytoene synthase (*PSY-1*), the key driver in carotenoid biosynthesis, exhibit altered kinetics of enzymes competing for the common precursor of carotenoids and tocopherols, i.e. GGPP, that induce changes in precursor/product ratios throughout the carotenoid pathway with no alteration in gene expression [36]. Furthermore, the perturbations in carotenoid content and precursor pools of *PSY-1*-overexpressing plants induce global changes in the metabolome outside the isoprenoid pathway, including the down-regulation of several flavonoids (e.g. NarCh and several flavonols).

Coupled changes in the phenylpropanoid and isoprenoid pathways have been demonstrated in various tomato mutants and transgenic plants. Transgenic tomato plants ectopically overexpressing *CHRYPTOCHROME2* displayed overproduction of anthocyanins and chlorophyll in their leaves, and of flavonoids and lycopene in their fruit [37]. The tomato fruit high-pigment (*hp*) mutants provide an additional example of the combined over-accumulation of flavonoids and isoprenoids, as fruit of these mutants show a simultaneous increase in the levels of flavonoids, chlorophylls, carotenoids, *α*-tocopherol and ascorbic acid [38],[39]. RNAi-mediated down-regulation of the *DE-ETIOLATED1* (*DET1*) gene (corresponding to the *hp-2* mutation) results in similar metabolic changes, including an increase in the levels of chlorogenic acid [40]. Overexpression of a *Vitis vinifera* R2R3-MYB transcription factor (*VvMYB5b*) in tomato induced pleiotropic changes, including down-regulation of phenylpropanoid metabolism and up-regulation of β-carotene [41]. Unlike previous reports describing a coupled increase in both pathways or opposite effects, in the *y* mutant studied here, we found coupled down-regulation of both pathways. This simultaneous effect on pathways generating antioxidant or photoprotective agents might reflect a mechanism linking the different classes of defense chemicals.

Cleavage of xanthophylls at the end of the carotenoid pathway produces the precursor for abscisic acid (ABA) [42]. ABA deficiency has been associated with elevated carotenoid content in tomato fruit of the *hp3*, *flacca* (*flc*) and *sitiens* (*sit*) mutants [43],[44], and in *ZEAXANTHIN EPOXIDASE* (*ZEP*)-silenced transgenic plants [44]. The *y* mutant displayed reduced detectable carotenoid levels, down-regulated expression of two carotenoid-related genes, including *NCED* encoding the enzyme catalyzing a committed step in ABA production, as well as down-regulation of *SlTHM27* and *SlMYB4-like*. The latter genes are orthologues of *AtMYB4* which has previously been reported to be involved in the UV-B response in an ABA-sensitive Arabidopsis mutant [45]. All of these results suggest that changes in ABA levels are likely to occur in the *y* mutant fruit. An analysis of ABA levels is therefore warranted in future studies with the *y* mutant.

### Down-Regulation of *SlMYB12* Underlies the *y* Mutant Phenotype

The extensive alterations revealed by transcriptomic and metabolomic analyses in the *y* mutant implied a deficiency in a regulatory factor rather than in a single, structural gene. Our analyses focused on tomato orthologues/homologues of particular members of the MYB and MYC (bHLH) family of transcription factors that have previously been associated with control of the phenylpropanoid and flavonoid pathways in other plant species. Four major lines of evidence support the conclusion that down-regulation of *SlMYB12* underlies the *y* mutant phenotype: (i) specific down-regulation of *SlMYB12* by the amiR RNA induces a *y* phenocopy; (ii) phenotype complementation was demonstrated upon overexpression of *SlMYB12* on the *y* genetic background; (iii) of the three putative flavonoid-related MYB-type transcription factors that are down-regulated in the *y* mutant, *SlMYB12* is the only one that maps to the region on tomato chromosome 1, which has previously been shown to harbor the *y* mutation [28]; (iv) the identification of an additional *SlMYB12* allele (*y-1*) that co-segregates with the colorless-peel phenotype among a large (>100 lines), unrelated introgression population.

A previous study demonstrated that the Arabidopsis *MYB12* controls flavonol biosynthesis mainly in the root, while its paralogue *AtMYB111* controls the biosynthesis of these metabolites primarily in cotyledons [20]. This is in accordance with our findings that alterations in the *y* mutant are also manifested in the root and fully expanded leaves, where *SlMYB12* is also expressed. Furthermore, the peel-associated expression pattern of *SlMYB12* is in accordance with the predominant accumulation of flavonoids in tomato fruit peel [2],[4],[5],[9]. The relatively high expression levels of *SlMYB12* at early stages of fruit development prior to flavonoid accumulation can account for early alterations in primary metabolites, such as organic and amino acids. Similar wide-range effects on target genes involved in both primary and secondary metabolism have been demonstrated for regulators of glucosinolate biosynthesis in Arabidopsis [46].

### The Regulatory Network Controlling Flavonoid Accumulation in Tomato Fruit Peel

*AtMYB12* was originally identified as a flavonol-specific transcription activator in Arabidopsis and like its orthologue in maize (factor P), it does not require a bHLH partner for promoter activation [18]. When expressed in tomato, *AtMYB12* activated flavonol biosynthesis as well as the caffeoylquinic acid biosynthetic pathway. The activity of *AtMYB12* expressed in tomato was suggested to mirror the function of the orthologous tomato protein MYB12 [23]. While overexpression of *AtMYB12* in tomato activates the caffeoylquinic acid biosynthetic pathway, down-regulation of *SlMYB12* in transgenic (cv. MT) plants also resulted in the accumulation of caffeic acid derivatives (e.g. dicaffeoylquinic acid III and tricaffeoylquinic acid). On the other hand, analysis of the *y* mutant (cv. AC background) did not reveal any significant changes in the levels of most caffeic acid derivatives, aside from the up-regulation of caffeic acid hexose IV. Furthermore, levels of several ferulic acid derivatives in a closely related branch of the pathway were significantly increased in the *y* mutant. Therefore, the up-regulation of these related side branches (i.e. caffeoylquinic acid and ferulic acid derivatives) upon both up- and down-regulation of the expression of the *MYB12* orthologue in tomato is more likely the result of a flux shift in the metabolic pathway than a direct outcome of altered transcriptional activation.

Stracke et al. [20] studied the functional redundancy and differential spatial expression characteristics of the R2R3-MYB factor subgroup 7 in Arabidopsis seedlings (AtMYB11, AtMYB12 and AtMYB111). They showed that all three members of this subgroup are flavonol-specific transcriptional regulators, and demonstrated that the final flavonol accumulation pattern is a result of the additive expression patterns of these three factors. Furthermore, MYB11, MYB12 and MYB111 displayed very similar target-gene specificity for several genes of flavonoid biosynthesis, including *AtCHS*, *AtCHI*, *AtF3H* and *AtFLS*. Our study revealed that in the fruit of both *y* and *35S:amiR-SlMYB12* plants, down-regulation of *SlMYB12* is accompanied by the suppression of *SlTHM27* (the tomato orthologue of *AtMYB4*) and *SlMYB4-like*. Both share a peel-associated expression profile during fruit development that is very similar to the pattern observed for *SlMYB12* (Figure S11). This suggests that *SlMYB12* is a direct activator of the *SlTHM27* and/or *SlMYB4-like* genes. Promoter-binding reporter assays need to be carried out to further test this suggestion.

To summarize, detailed characterization of the tomato colorless-peel *y* mutant led to the elucidation of a transcriptional network involving SlMYB12, which controls the accumulation of NarCh in tomato fruit cuticle. Furthermore, it demonstrated that a lack of NarCh in the tomato fruit cuticle is associated with alterations in other characteristics, such as thickness, elasticity, cutin content and wax composition, and that in contrast to previous suggestions, NarCh is not involved in water transport across the fruit cuticle. Most previous studies on the outer layer of plants have been largely limited to the vegetative organs and have focused on the formation of cuticular lipids. Our study provides valuable insight into the surface of a reproductive organ. It sheds further light on the biosynthesis of fruit epidermis flavonoids, another class of cuticle constituents that are present in many plant species and play a prominent role in the association between structure and function of this essential surface layer.

## Materials and Methods

### Plant Material

Seeds from homozygous *y* mutant plants (LA3189) in the cv. Ailsa Craig (AC) background, as well as from wt cv. AC, were obtained from the Tomato Genetics Resource Center (TGRC; http://tgrc.ucdavis.edu). Flowers of greenhouse-grown plants were marked at anthesis, and fruit were harvested according to appearance and days post-anthesis (DPA): ∼25 DPA - Immature Green, ∼42 DPA - Mature Green, ∼44 DPA - Breaker, ∼46 DPA - Orange and ∼48 DPA - Red. Each biological repeat was a mixture of four to five individual fruits from the same stage of development. Immediately upon harvesting, peel and flesh (without the gel and seeds) were manually dissected and frozen in liquid nitrogen.

### Confocal Microscopy

Confocal microscopy was carried out on small pieces of isolated cuticles from the wt and *y* mutant mounted on microscope slides, using a Leica TCS NT confocal laser scanning microscope (Leica, Heidelberg, Germany) with excitation beam splitter DD 488/568.

### Flavonoid Detection

Diphenyl boric acid 2-amino-ethyl ester (DPBA, also called Naturstoff A) was used for the staining of free 3′, 4′ and/or 5′OH groups including non-glycosylated flavonoids. In initial analysis, untreated fruit slices and peel pieces were photographed on a UV (312 nm) transilluminator. Subsequently, fruit slices and peel pieces were placed for 2 h in a saturated solution (<0.5% w/v) of DPBA (Sigma) with 0.01% Triton X-100 and rephotographed on the UV transilluminator [47],[48]. Stained non-glycosylated flavonoids were colored in red while non-stained samples reflected only white illumination.

### Generation of Constructs and Plant Transformation

The full-length *SlCHS1* gene was isolated from genomic DNA of cv. AC fruit by PCR amplification using the primers listed in Table S3, sub-cloned into pFLAP100 containing the CaMV 35S promoter and then cloned into the binary pBINPLUS vector [49]. The amiR*-MYB12* (artificial microRNA targeting MYB12) synthetic gene was synthesized by Bio S&T (Montreal, Canada) and cloned with the CaMV 35S promoter into the pBINPLUS binary vector. Cotyledon transformation in cv. Micro Tom (MT) tomato was performed according to Dan et al. [50].

### Gene-Expression Analysis

For array analysis, total RNA was extracted by the hot phenol method [51] from tissues pooled from three to four fruits from either three of the developmental stages examined. The cDNA synthesized by the Invitrogen Superscript II RTase was used as a template to generate biotinylated cRNA that was fragmented and hybridized to the Affymetrix GeneChip Tomato Genome Array as described in the Affymetrix technical manual (available at www.affymetrix.com), with two biological replicates for the *y* mutant and three biological replicates for the wt. Replicate reproducibility and variance filtering procedures were carried out on wt transcript expression data as previously described in Mintz-Oron et al. [9]. Normalization of log2-based expression-intensity values was carried out using RMA analysis [52] implemented by the R microarray analysis package (http://www.R-project.org). Initial filtering of the genes was performed using the absent/present call acquired by MAS5 analysis software (Affymetrix 2002). Transcripts with at least one stage containing a present call were retained. Next, all expression values below the 10th percentile were set to the 10th percentile value. Transcripts with poor-quality spots showing low replicate reproducibility (high RSD) in at least a third of the tested stages were eliminated from further analysis. Differential *y* mutant and wt transcripts were defined as those having at least twofold intensity ratio in *y* vs. wt at at least one developmental stage of one of the tested fruit tissues (peel or flesh). RT-PCR analysis was carried out as described in Mintz-Oron et al. [9]. Primer sequences designed by Express software (Applied Biosystems) are provided in Table S3 and Table S5.

### Non-Targeted UPLC-QTOF-MS Profiling of Semi-Polar Compounds and Data Analysis

Non-targeted analysis of semi-polar compounds was carried out with an UPLC-QTOF instrument (Waters Premier; Waters Chromatography, Milford, MA, USA), with the UPLC column connected online to a UV detector and then to the MS detector as previously described [6]. Separation of metabolites was performed by gradient elution (acetonitrile-water, containing 0.1% formic acid) on a 100×2.1-mm i.d., 1.7-µm UPLC BEH C18 column (Waters Acquity). Masses of the eluted compounds (m/z range from 50 to 1500 Da) were detected with a QTOF-MS equipped with an ESI source (performed in both positive and negative modes). XCMS data processing [53] was carried out as previously described [9].

PCA of metabolic profiles was performed on data sets obtained as XCMS output with the MATLAB Statistical Toolbox. The PCA plot presented in Figure 9E was constructed with the software package TMEV [54], and data were pretreated by normalization to the median of the entire sample set for each mass signal and log_10_ transformation. Metabolites that differed between *y* and wt fruit tissues were detected at the breaker, orange and red stages of development. To ensure data robustness to statistical analysis, mass signals for which the maximal intensity across all samples was <50 units for peel samples or <40 units for flesh samples (arbitrary units proportional to peak area calculated by XCMS) were discarded. Statistical filtering was carried out on the mass signals to identify differential markers between the wt and *y* mutant. The remaining peaks were those positive for the following filter at at least one of the three developmental stages:
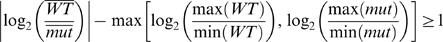
i.e. fold change between the means of wt and mutant had to be at least twice as high as the maximal fold change within the repeats of either *y* mutant or wt. Mass signals of interest retained after the filtering stage were assigned to metabolites using automatic assignment of all mass signals to metabolites by clustering. To cluster together masses belonging to the same metabolite, a custom computer program implemented in MATLAB 7.3 was developed. The program accepts as input the filtered intensity data following XCMS analysis and the chromatographic retention time of each marker. A matrix is calculated that describes the distance between all pairs of mass signals based on Spearman correlation among their intensities across all samples and differences in their retention times. Therefore: distance Dij between two markers i and j with retention times RTi and RTj and Spearman correlation coefficient ρij between their intensities across all samples was defined as: 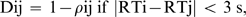 otherwise:
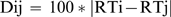



A hierarchical average linkage clustering algorithm was applied to the distance matrix to define mass signals to metabolite assignments. The clustering distance cutoff was set to 0.34. The cutoff was determined by maximization of the similarity assessed by the Jaccard similarity coefficient of the clustering results to the test set containing manual assignment of mass signals to 26 metabolites. The Jaccard similarity coefficient is defined as:
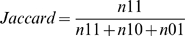
where for each pair of mass signals from the automatically clustered set and the manually curated assignment set:

n11 is the amount of pairs assigned to the same metabolite both automatically and manually

n10 is the amount of pairs assigned to the same metabolite manually, but not automatically

n01 is the amount of pairs assigned to the same metabolite automatically, but do not belong to the same metabolite in the manual assignment

### Analysis of GC-MS Data

MS/MS was performed on manually selected molecular ions of differential metabolites with high intensity. Putatively assigned differential compounds and compounds previously detected in tomato in our lab were added to Table S2, which contains names, elemental composition, MS/MS fragments, UV absorbance, etc. (in total 71 compounds). The intensity values for these compounds resulting from XCMS analysis were manually checked and reintegrated in cases of problematic peaks.

### GC-MS Profiling of Derivatized Polar Extracts and Data Analysis

GC-MS analysis of polar metabolites in the *y* mutant and wt fruit tissues samples (n = 3) was carried out as previously described [9]. For PCA plotting, the data were pretreated as follows: missing values for metabolites in one of the three replicates were exchanged for the average between the replicates, zero values were replaced by a value 10 times lower than the minimal non-zero value in the data set, data were normalized to the mean of each metabolite across all samples and log-transformed. For statistical analysis of the whole data matrix, a two-way ANOVA test was performed with the two discriminating factors being genotype (wt or *y*) and fruit developmental stage. Multiple hypothesis control was carried out by an FDR procedure with the Q-value set to 0.05. For all metabolites that showed a significant change, post-hoc analysis was carried out to determine the stage at which the change occurred. The significance of the difference between the triplicate repeats of wt values and the triplicate repeats of *y* values was assessed as follows: i) when two or more values were non-quantifiable in one of the genotypes the comparison was not carried out, ii) in the case of a triplet of zeros (compound not detected in sample) vs. another triplet of zeros, the *P*-value was set to 1, iii) for all other cases, a two-sided contrast *t*-test was carried out with the variance estimated from the mean squared error from a two-way ANOVA test.

### Isoprenoid Extraction and Analysis

Isoprenoid extraction was performed as described by Fraser et al. [55] and by Bino et al. [39], with several modifications: frozen tomato powder (0.5 g) was extracted with 2.4 ml methanol containing 0.1% butylated hydroxytoluene (BHT) and 100 µl *trans*-β-apo-8-carotenal (internal standard, 100 µg ml^−1^). The samples were shaken for 5 min, then 2.5 ml Tris-HCl buffer pH 7.5 (50 mM) was added (containing 1 M NaCl) and samples were shaken for 10 min before adding 2 ml of cold chloroform, shaking for 10 min and centrifuging. The aqueous phase was re-extracted with 1 ml cold chloroform and the chloroform fractions were combined, dried under a stream of nitrogen gas and resuspended in 0.5 ml ethylacetate. The samples were prepared under low light at 4°C. The HPLC system consisted of a 2690 separation module, a 2996 Photo Diode Array detector, and a 470 scanning fluorescence detector connected online (Waters). A YMC-Pack reverse-phase C30 column (250×4.6 mm; 5 µm), coupled to a 4×3 mm C18 guard (Phenomenex) (maintained at 30°C) was used. The mobile-phase composition, gradient and flow rate were as described by Fraser et al. [55]. The UV spectra were monitored from 200 to 750 nm. The fluorescence detector, set at 296 nm excitation and 340 nm emission, was used for the analysis of tocopherols. Data were collected and analyzed using the Millennium32 software (Waters). Absorbance spectra and retention times of eluting peaks were compared with those of commercially available isoprenoid standards: *δ*-tocopherol and *γ*-tocopherol (Supelco), *α*-tocopherol (Aldrich), chlorophylls a and b and lutein (Fluka), *trans*-β-carotene and *trans*-lycopene (Sigma). Phytoene, phytofluene and *ζ*-carotene were putatively identified by comparison of their absorbance spectra and retention times to the data presented by Fraser et al. [55]. Peak areas of the compounds were determined at the wavelength providing maximum absorbance (Table S5).

### Cuticular Wax and Cutin Analyses

For wax and cutin analyses, the cuticle was first isolated from manually dissected peels as described by Hovav et al. [56]. Cuticular waxes were extracted by immersing the peels (30–40 mg per sample) twice for 1 to 2 h in 5 ml of CHCl_3_ at room temperature. Both solutions were combined and n-tetracosane was added as an internal standard. The solvent was removed under a gentle stream of nitrogen; the remaining wax mixture was redissolved in 200 ml of CHCl_3_ and stored at 4°C until use. After the wax extraction, the remaining cutin samples were depolymerized by the addition of 2 ml of 1 M BF_3_/methanol for 3 h at 70°C, and n-tetracosane was added as an internal standard. Then, after the addition of 2 ml water, the cutin monomer mixture was extracted three times with diethylether. The solvent of the combined extracts was removed under nitrogen; the remaining cutin mixture was redissolved in 1 ml of CHCl_3_ and stored at 4°C until use. A 50-µl aliquot of the wax or cutin samples was used for independent GC analyses. CHCl_3_ was evaporated from the samples under nitrogen while heating to 50°C. Then, the wax or cutin mixtures were treated with bis-N,N-(trimethylsilyl)trifluoroacetamide (BSTFA, Sigma-Aldrich) in pyridine (30 min at 70°C) to transform all hydroxyl-containing compounds into the corresponding trimethylsilyl derivatives. The qualitative composition of the resulting mixtures was studied with capillary GC (5890N, Agilent; 30 m HP-1, 0.32 mm i.d., df = 1 µm) with He carrier gas inlet pressure adjusted for a constant flow of 1.4 ml min^−1^ and MS detector (5971, Agilent). GC was carried out with temperature-programmed on-column injection at 50°C, oven 2 min at 50°C, raised by 40°C min^−1^ to 200°C, held for 2 min at 200°C, raised by 3°C min^−1^ to 320°C and held for 30 min at 320°C. The quantitative composition of the mixtures was studied using capillary GC with FID under the same GC conditions as described above, but with H_2_ carrier gas inlet pressure programmed for a constant flow of 2 ml min^−1^. Single compounds were quantified against the internal standard by manual integration of peak areas.

### Mechanical Tests

The mechanical properties of isolated cuticles were measured as previously described by Matas et al. [57], using an extensometer equipped with a linear displacement transducer (Mitutoyo, Kawasaki, Japan) that was customized to work with the corresponding isolated cuticle samples (resolution of ±1 µm).

### Gene Reconstruction and Assignment of Chromosomal Locations

Full-length cDNA sequences of *SlJAF13*, *SlMYB12-like*, *SlMYB12*, *SlMYB4-like*, *SlANT2*, *SlMYB111* and *SlMYB61* were reconstructed by bioinformatics and RACE analyses using the Sequencher 4.7 software (GeneCodes Corporation) and Clontech SMART™ RACE kit. Full-length ORFs of tomato chalcone synthase-1 (*CHS1*; TC170658), chalcone synthase-2 (*CHS2*; TC172191), and seven putative tomato flavonoid-related transcription factors (*SlJAF13*, *SlMYB12*, *SlMYB4-like*, *SlTHM27*, *SlANT2*, *SlMYB111* and *SlMYB61*) were PCR-amplified from cDNA of *y* mutant and wt fruit peels by the specific oligonucleotides listed in Table S3. The amplified PCR products were sequenced and analyzed by the Sequencher software. The reconstructed *SlJAF13*, *SlMYB4-like* and *SlMYB12-like* mapped to the following *Solanum lycopersicum* NCBI genomic clones: AP009298 (chromosome 8), AC209513 (chromosome 6) and AC216658 (chromosome 6), respectively. The reconstructed *SlMYB12, SlTHM27* and *SlMYB111* were assigned to tomato chromosomes 1, 10 and 11, respectively, by the tomato inter-specific ILs [58].

## Supporting Information

Figure S1 Flavonoid staining of fruit cuticles, water-transpiration tests and cell-wall related phenotypes of post-harvest fruit. (A) Confocal microphotographs (excitation at 488 nm) of isolated fruit cuticles at the red stage of development, demonstrating the lack of flavonoids in the *y* mutant cuticle. (B) Placing of diphenylboric acid 2-aminoethyl ester (DPBA) stained isolated fruit cuticles from red stage of fruit development over a UV light table reveals red staining of wt cuticles due to the presence of flavonoids, while the *y* mutant isolated cuticles reveals no staining (white). Con. Control, unstained isolated cuticles. (C) Cuticles (disks of 1.5 cm diameter) were isolated by cellulose and pectinase according to Lopez-Casado et al. [57]. Isolated cuticles were mounted to stainless steel transpiration chambers filled with 800 µl distilled water and water loss across the cuticle was measured by gravimetry as described in detail by Schreiber et al. [Schreiber L, Elshatshat S, Koch K, Lin J, Santrucek J (2006). AgCl precipitates in isolated cuticular membranes reduce rates of cuticular transpiration. Planta 223: 283–90]. Permeance (m/s) was calculated from linear regression lines fitted to the transpiration kinetics (weight loss vs. time). For each developmental state of the *y* mutant and the wt, cuticular transpiration was measured for 20 individual cuticular membranes. Results are given as means with 95% confidence intervals. Statistical significant differences were tested applying a t-test. (D) Water loss of whole post harvest fruit. Abbreviations: Br - breaker, Or - orange, Re - re. (E) Post-harvest phenotypes of wt and *y* mutant fruit indicate differences in cell wall degradation. While the shrunken and wrinkled but relatively firm wt fruit half sinks in water as one piece, the peel of the smooth, non-shrunken but soft and hollow *y* mutant half separates from its degrading inner tissues.
(9.04 MB PPT)

Figure S2 *SlCHS* expression and co-suppression. (A) RT-PCR relative expression analysis, in wt and *y* fruit tissues, of *SlCHS1* (TC170658) and *SlCHS2* (TC172191) transcripts reveal down-regulation of these two transcripts during the three tested stages of fruit developmental (n = 3; P<0.05; bars represent standard error). (B) Sectorial co-suppression of NarCh accumulation in over-expressing transgenic line, cv. MT. (C) Whole fruit and peels of *SlCHS1* over-expressing line results in pink color fruit, which do not accumulate the yellow NarCh in their peel. Br - breaker, Or - orange, Re - red.
(0.87 MB PPT)

Figure S3 Gene-expression profile clusters. Four hundred and six *y* and wt differentially expressed transcripts were clustered into 40 expression profiles (divided into 20 peel and 20 flesh clusters; see Table S1). Expression profile hierarchical clustering was performed on the normalized (mean-center) log2-based values of the *y* mutant and wt differential transcripts, using average linkage clustering method with Pearson correlation distance measure (implemented in MATLAB, version 7.3.0, TheMathWorks).
(0.19 MB PPT)

Figure S4 PCA of metabolic profiles obtained by UPLC-QTOF-MS analysis, including samples of wt and *y* peel and flesh tissues along five stages of the fruit development (n = 3).
(0.04 MB PPT)

Figure S5 Alterations in metabolite and gene-expression levels in the phenylpropanoid pathway as detected in the *y* mutant fruit flesh tissue. (A) Changes in gene expression and metabolite levels in tomato fruit flesh (detected as described for Figure 5). Red and blue colors represent up or down regulation, respectively. (B) Real Time-PCR expression analyses of selected transcripts from the phenylpropanoids pathway in wt and *y* mutant tomato flesh tissues at the breaker stage of fruit development. Indicated by asterisks are significant differences analyzed by a student's t-test (n = 3; P<0.05; bars indicate standard errors). Gene identifiers and RT-PCR primers are listed in Table S3. #, see Figure 5.
(0.20 MB PPT)

Figure S6 The *y* mutation affects metabolism and gene expression in plant organs other than fruit. RT-PCR expression analyses of selected phenylpropanoid/flavonoid-related transcripts (trans; full gene names are listed in table S3) in: (A) young leaves. (B) fully expanded leaves. Indicated by asterisks are significant differences analyzed by student's t-test (n = 3; P<0.05; bars indicate standard errors). Gene identifiers and primers are listed in Table S3. (C) PCA of metabolic profiles obtained by UPLC-QTOF-MS analysis clearly distinguish between samples of wt and *y* mutant roots.
(0.06 MB PPT)

Figure S7 Cuticular wax composition in the *y* mutant and wt fruit peel at three tested stages of fruit development, as analyzed by GC-MS/FID. Wax constituents are sorted by compound classes according to carbon number in the chains (n = 5, error bars indicate standard errors).
(0.32 MB PPT)

Figure S8 *SlMYB12* assignment to tomato chromosome 1. (A) *BstBI* digestion of *SlMYB12* genomic fragments amplified from the tomato set of interspecific introgression lines between cv. M82 and *Lycopersicon pennellii* [58]. (B) The Il1-1 *pennellii* chromosome segment, which does not overlap with other introgression lines resides between 17 CM to 41 CM.
(0.31 MB PPT)

Figure S9 Multiple alignment of flavonoid-related MYB transcription factors and the *SlMBY12* alleles. (A) Multiple alignment of the flavonoid related factors that were down-regulated in the *y* mutant (tomato THM27/MYB4, MYB4-like and MYB12 putative protein products of TC174616 and other gene sequences that were reconstructed in this study), their closest paralogue (putative protein product of *SlMYB12-like* that was reconstructed in this study) and protein products of their Arabidopsis orthologues (MYB11 and MYB12, NP_191820 and NP_182268, respectively) as well as the predicted protein product of the *SlMYB12* allele, *y-1*. Amino acid (aa) substitutions and the aa deletion in the *y-1* allele are indicated in pink. (B) Nucleic acids alignment of the two *SlMYB12* alleles. Exonic and intronic sequence differences are highlighted in grey. Red asterisks and underlines indicate premature polyadenylation (pad) sites of the *y-1* transcripts. Out of 20 sequenced RACE products 4, 4, 3, 4, 3 were pad1a, pad2a, pad3a, pad4a, pad5a, pad6a versions and the short wt version, respectively.
(0.09 MB PPT)

Figure S10 Total Ion Chromatograms of *y*, cv. AC, *35S:amiR-SlMYB12* and cv. MT peel samples at the red stage of fruit development, acquired in negative mode by the UPLC-QTOF-MS instrument. Putative identifications of the differential compounds are: 1- quercetin-dihexose-deoxyhexose, 2- quercetin-hexose-deoxyhexose-pentose, 3- quercetin rutinoside (rutin), 4- phloretin-di-C-hexose, 5- kaempferol-glucose-rhamnose, 6- naringenin chalcone, 7- dicaffeoylquinic acid III, 8- tricaffeoylquinic acid. Red and blue numbers indicate metabolites that showed elevated or reduced levels in the mutant/transgene samples compared to those of their corresponding wt. Black numbers indicate metabolites that did not differ between the *y* mutant and its corresponding cv. AC wt.
(0.16 MB PPT)

Figure S11 RT-PCR relative transcript expression analyses of *SlTHM27* (previously described by Mintz-Oron et al. [9], *SlMYB4-like* and *SlMYB12-like* in wt peel and flesh during five stages of fruit development.
(0.06 MB PPT)

Table S1 All transcripts identified by the microarray analysis as differentially expressed between the *y* mutant and wt fruit tissues.
(0.20 MB XLS)

Table S2 List of putative metabolites identified in tomato fruit peel and flesh by UPLC-QTOF-MS analysis.
(0.13 MB XLS)

Table S3 Summary of phenylpropanoid/flavonoid-related transcripts.
(0.12 MB DOC)

Table S4 Summary of isoprenoid-related transcripts.
(0.05 MB DOC)

Table S5 Targeted HPLC-PDA isoprenoid analysis.
(0.04 MB DOC)
