# Supplementary material for: Fruit-Surface Flavonoid Accumulation in Tomato Is Controlled by a SlMYB12-Regulated Transcriptional Network
Source: PLoS Genet. 2009 Dec 18;5(12):e1000777. doi: 10.1371/journal.pgen.1000777 (PMC2788616; doi:10.1371/journal.pgen.1000777)
Supplement: Figure S1 — Flavonoid staining of fruit cuticles, water-transpiration tests and cell-wall related phenotypes of post-harvest fruit. (A) Confocal microphotographs (excitation at 488 nm) of isolated fruit cuticles at the red stage of development, demonstrating the lack of flavonoids in the y mutant cuticle. (B) Placing of diphenylboric acid 2-aminoethyl ester (DPBA) stained isolated fruit cuticles from red stage of fruit development over a UV light table reveals red staining of wt cuticles due to the presence of flavonoids, while the y mutant isolated cuticles reveals no staining (white). Con. Control, unstained isolated cuticles. (C) Cuticles (disks of 1.5 cm diameter) were isolated by cellulose and pectinase according to Lopez-Casado et al. [57]. Isolated cuticles were mounted to stainless steel transpiration chambers filled with 800 µl distilled water and water loss across the cuticle was measured by gravimetry as described in detail by Schreiber et al. [Schreiber L, Elshatshat S, Koch K, Lin J, Santrucek J (2006). AgCl precipitates in isolated cuticular membranes reduce rates of cuticular transpiration. Planta 223: 283–90]. Permeance (m/s) was calculated from linear regression lines fitted to the transpiration kinetics (weight loss vs. time). For each developmental state of the y mutant and the wt, cuticular transpiration was measured for 20 individual cuticular membranes. Results are given as means with 95% confidence intervals. Statistical significant differences were tested applying a t-test. (D) Water loss of whole post harvest fruit. Abbreviations: Br - breaker, Or - orange, Re - re. (E) Post-harvest phenotypes of wt and y mutant fruit indicate differences in cell wall degradation. While the shrunken and wrinkled but relatively firm wt fruit half sinks in water as one piece, the peel of the smooth, non-shrunken but soft and hollow y mutant half separates from its degrading inner tissues. (9.04 MB PPT) [file pgen.1000777.s001.ppt]

## Slide 1
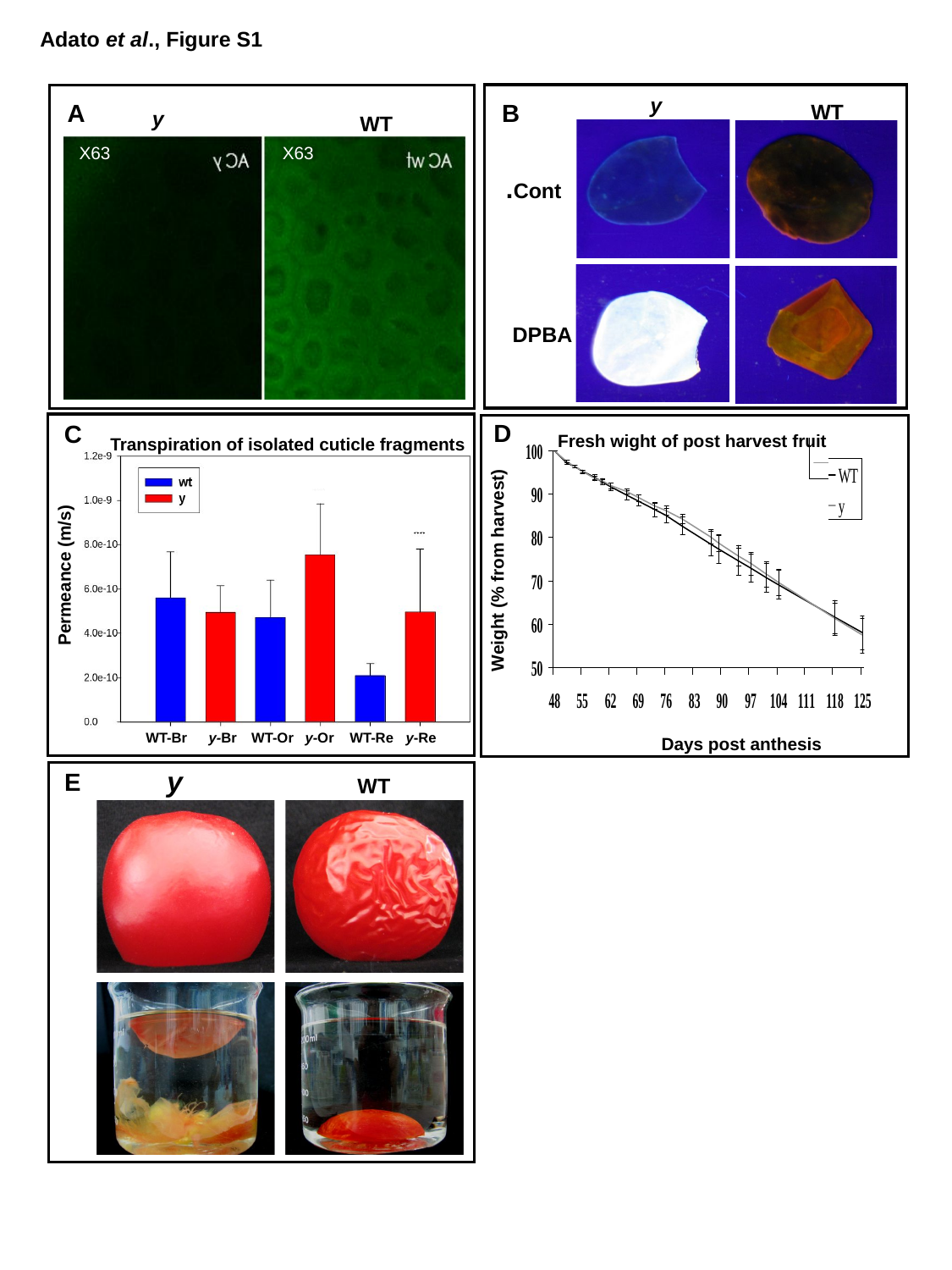

Adato et al., Figure S1
y
B
WT
Cont.
DPBA
A
y
WT
X63
X63
D
Fresh wight of post harvest fruit
Weight (% from harvest)
Days post anthesis
C
Transpiration of isolated cuticle fragments
Permeance (m/s)
WT-Br
y-Br
WT-Or
y-Or
WT-Re
y-Re
y
E
WT
