# Supplementary material for: Fruit-Surface Flavonoid Accumulation in Tomato Is Controlled by a SlMYB12-Regulated Transcriptional Network
Source: PLoS Genet. 2009 Dec 18;5(12):e1000777. doi: 10.1371/journal.pgen.1000777 (PMC2788616; doi:10.1371/journal.pgen.1000777)
Supplement: Figure S2 — SlCHS expression and co-suppression. (A) RT-PCR relative expression analysis, in wt and y fruit tissues, of SlCHS1 (TC170658) and SlCHS2 (TC172191) transcripts reveal down-regulation of these two transcripts during the three tested stages of fruit developmental (n = 3; P<0.05; bars represent standard error). (B) Sectorial co-suppression of NarCh accumulation in over-expressing transgenic line, cv. MT. (C) Whole fruit and peels of SlCHS1 over-expressing line results in pink color fruit, which do not accumulate the yellow NarCh in their peel. Br - breaker, Or - orange, Re - red. (0.87 MB PPT) [file pgen.1000777.s002.ppt]

## Slide 1
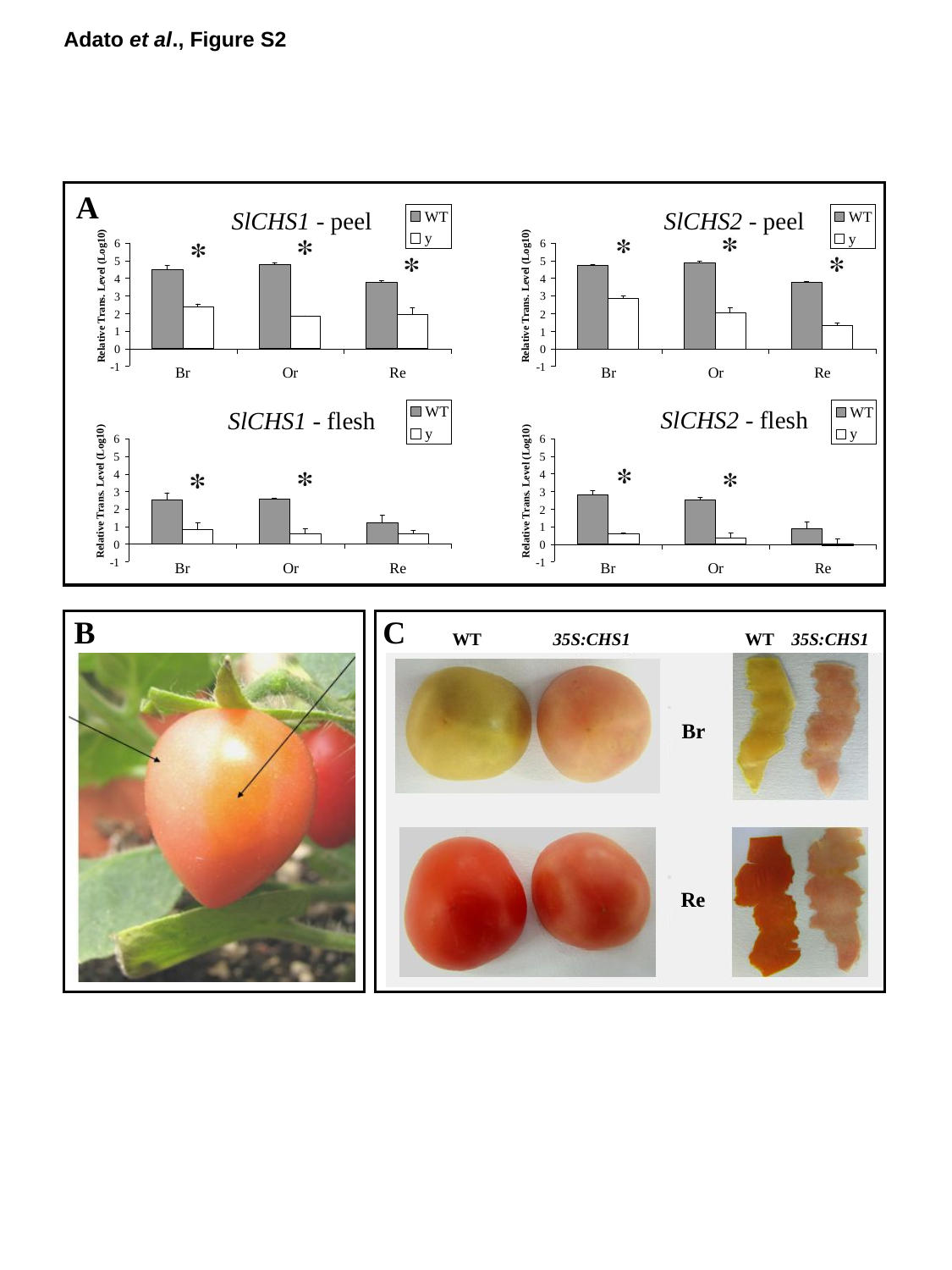

Adato et al., Figure S2
A
SlCHS2 - peel
SlCHS1 - peel
Relative Trans. Level (Log10)
Relative Trans. Level (Log10)
SlCHS2 - flesh
SlCHS1 - flesh
Relative Trans. Level (Log10)
Relative Trans. Level (Log10)
B
C
WT
35S:CHS1
WT
35S:CHS1
Br
Re
