# Supplementary figures and images for: Fruit-Surface Flavonoid Accumulation in Tomato Is Controlled by a SlMYB12-Regulated Transcriptional Network
Source: PLoS Genet. 2009 Dec 18;5(12):e1000777. doi: 10.1371/journal.pgen.1000777 (PMC2788616; doi:10.1371/journal.pgen.1000777)

## Slide 1
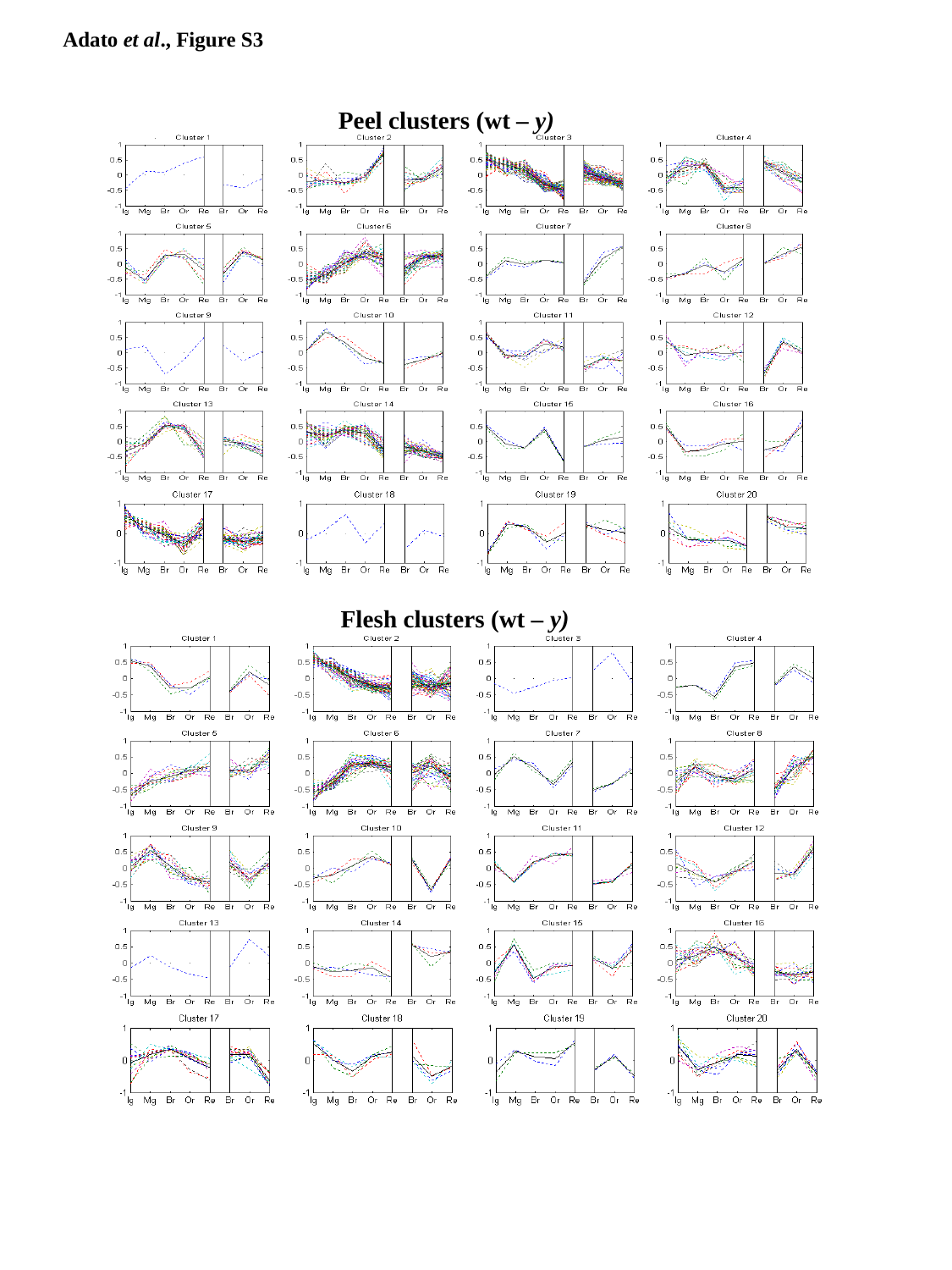

Adato et al., Figure S3
Peel clusters (wt – y)
Flesh clusters (wt – y)

Supplement: Figure S3 — Gene-expression profile clusters. Four hundred and six y and wt differentially expressed transcripts were clustered into 40 expression profiles (divided into 20 peel and 20 flesh clusters; see Table S1). Expression profile hierarchical clustering was performed on the normalized (mean-center) log2-based values of the y mutant and wt differential transcripts, using average linkage clustering method with Pearson correlation distance measure (implemented in MATLAB, version 7.3.0, TheMathWorks). (0.19 MB PPT) [file pgen.1000777.s003.ppt]

## Slide 1
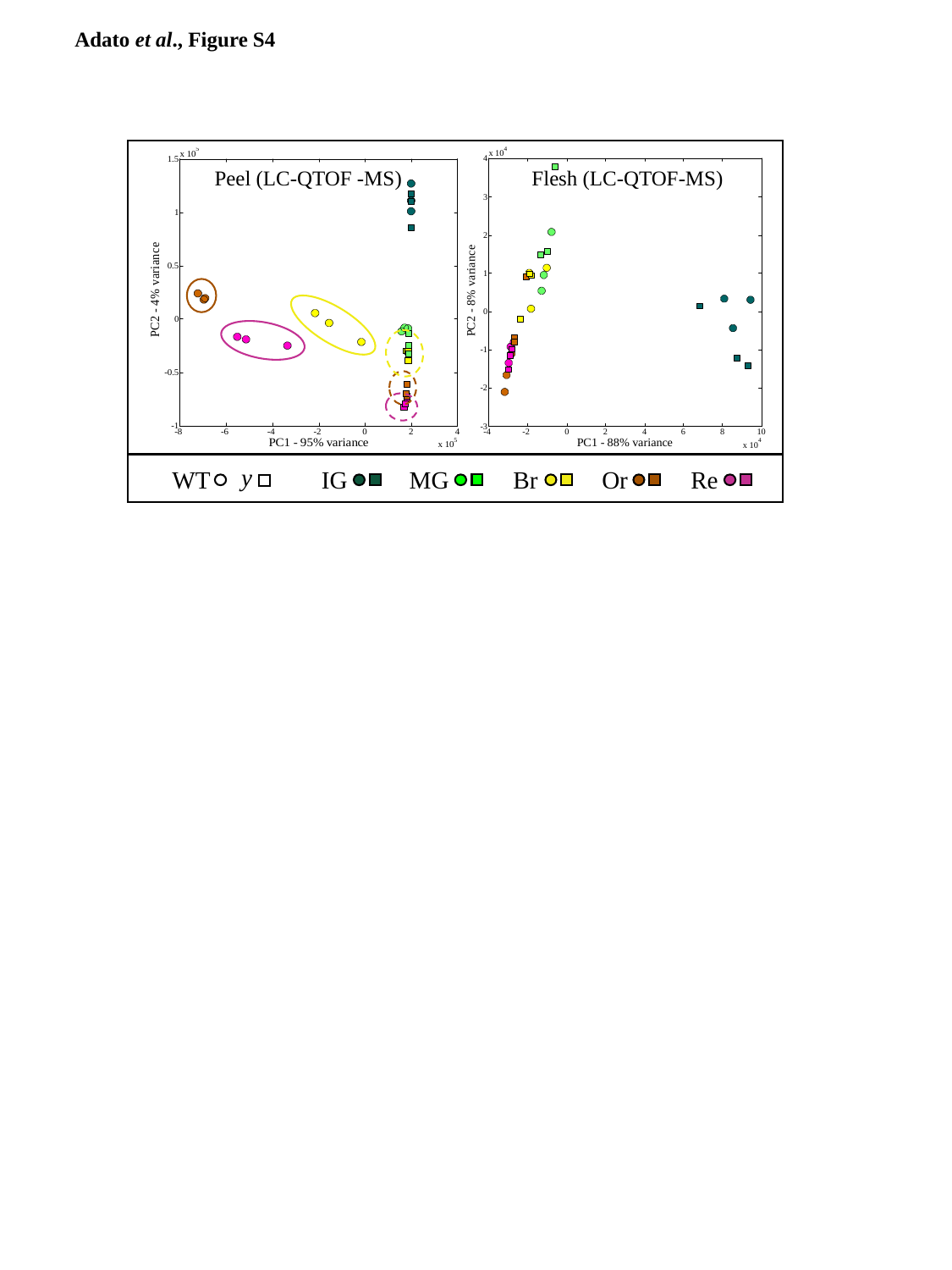

Adato et al., Figure S4
Peel (LC-QTOF -MS)
Flesh (LC-QTOF-MS)
y
WT
IG
MG
Br
Or
Re

Supplement: Figure S4 — PCA of metabolic profiles obtained by UPLC-QTOF-MS analysis, including samples of wt and y peel and flesh tissues along five stages of the fruit development (n = 3). (0.04 MB PPT) [file pgen.1000777.s004.ppt]
