# Supplementary material for: Fruit-Surface Flavonoid Accumulation in Tomato Is Controlled by a SlMYB12-Regulated Transcriptional Network
Source: PLoS Genet. 2009 Dec 18;5(12):e1000777. doi: 10.1371/journal.pgen.1000777 (PMC2788616; doi:10.1371/journal.pgen.1000777)
Supplement: Figure S5 — Alterations in metabolite and gene-expression levels in the phenylpropanoid pathway as detected in the y mutant fruit flesh tissue. (A) Changes in gene expression and metabolite levels in tomato fruit flesh (detected as described for Figure 5). Red and blue colors represent up or down regulation, respectively. (B) Real Time-PCR expression analyses of selected transcripts from the phenylpropanoids pathway in wt and y mutant tomato flesh tissues at the breaker stage of fruit development. Indicated by asterisks are significant differences analyzed by a student's t-test (n = 3; P<0.05; bars indicate standard errors). Gene identifiers and RT-PCR primers are listed in Table S3. #, see Figure 5. (0.20 MB PPT) [file pgen.1000777.s005.ppt]

## Slide 1
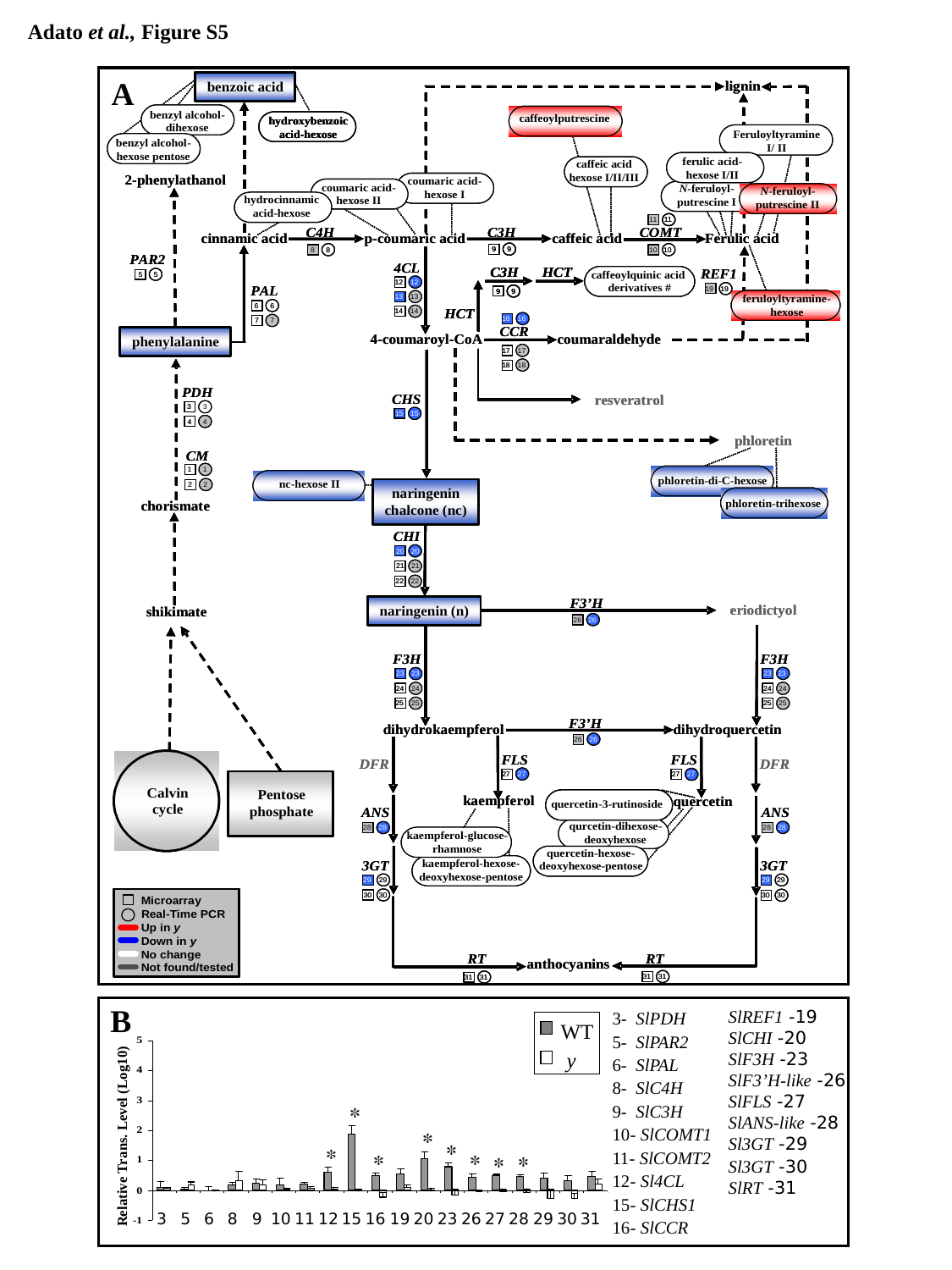

Adato et al., Figure S5
A
#
B
3- SlPDH
5- SlPAR2
6- SlPAL
8- SlC4H
9- SlC3H
10- SlCOMT1
11- SlCOMT2
12- Sl4CL
15- SlCHS1
16- SlCCR
19- SlREF1
20- SlCHI
23- SlF3H
26- SlF3’H-like
27- SlFLS
28- SlANS-like
29- Sl3GT
30- Sl3GT
31- SlRT
WT
y
Relative Trans. Level (Log10)
3
5
6
10
11
12
15
16
19
20
23
26
27
28
29
30
8
9
31
