# Supplementary material for: Fruit-Surface Flavonoid Accumulation in Tomato Is Controlled by a SlMYB12-Regulated Transcriptional Network
Source: PLoS Genet. 2009 Dec 18;5(12):e1000777. doi: 10.1371/journal.pgen.1000777 (PMC2788616; doi:10.1371/journal.pgen.1000777)
Supplement: Figure S6 — The y mutation affects metabolism and gene expression in plant organs other than fruit. RT-PCR expression analyses of selected phenylpropanoid/flavonoid-related transcripts (trans; full gene names are listed in table S3) in: (A) young leaves. (B) fully expanded leaves. Indicated by asterisks are significant differences analyzed by student's t-test (n = 3; P<0.05; bars indicate standard errors). Gene identifiers and primers are listed in Table S3. (C) PCA of metabolic profiles obtained by UPLC-QTOF-MS analysis clearly distinguish between samples of wt and y mutant roots. (0.06 MB PPT) [file pgen.1000777.s006.ppt]

## Slide 1
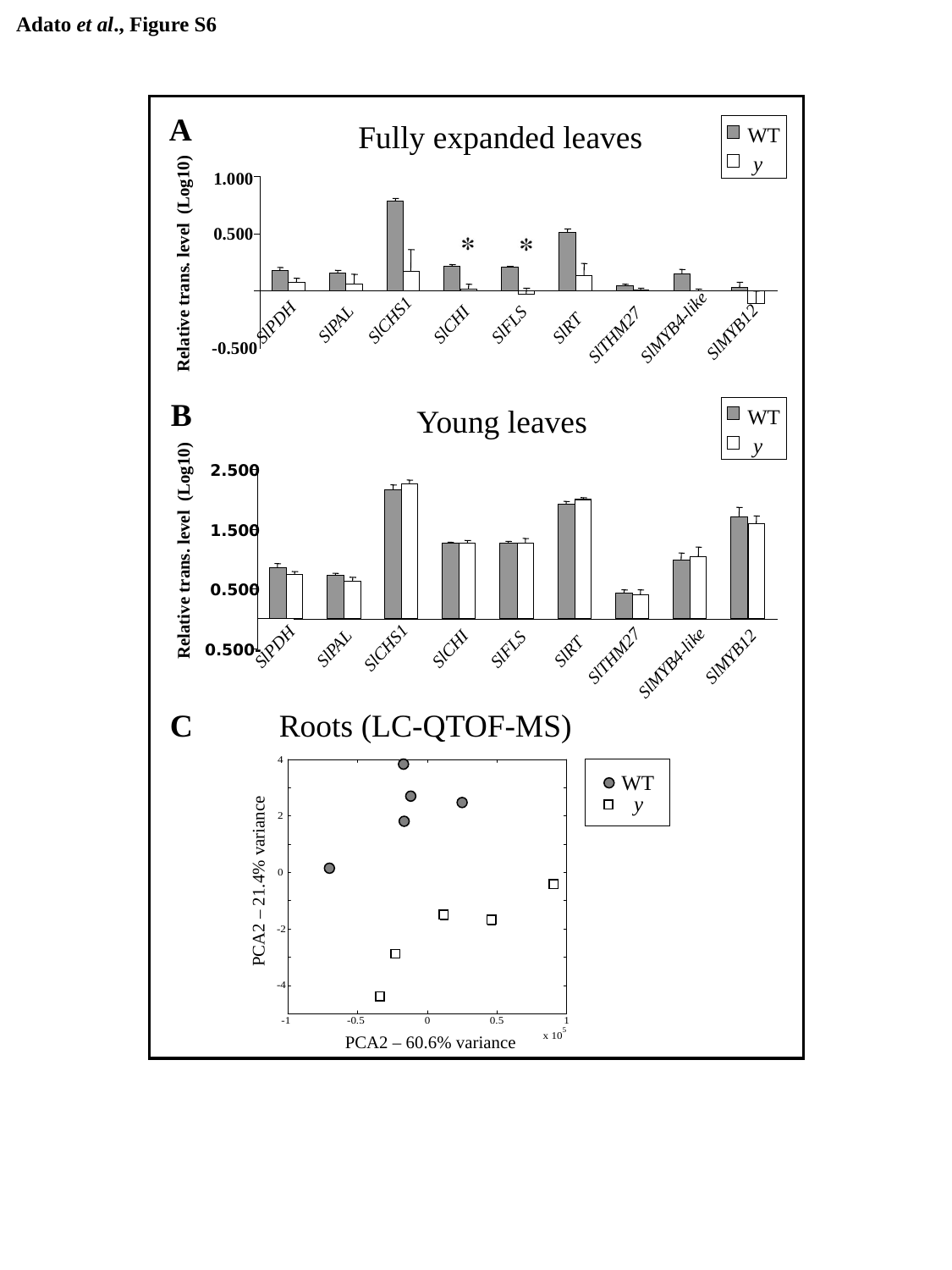

Adato et al., Figure S6
W.T.
A
Fully expanded leaves
WT
y
y
1.000
0.500
Relative trans. level (Log10)
SlCHS1
SlPDH
SlPAL
SlCHI
SlFLS
SlMYB4-like
SlRT
SlMYB12
SlTHM27
-0.500
B
Young leaves
WT
y
2.500
1.500
0.500
-0.500
Relative trans. level (Log10)
SlPDH
SlCHS1
SlPAL
SlCHI
SlFLS
SlRT
SlTHM27
SlMYB12
SlMYB4-like
C
Roots (LC-QTOF-MS)
WT
y
PCA2 – 21.4% variance
PCA2 – 60.6% variance
