# Supplementary material for: Fruit-Surface Flavonoid Accumulation in Tomato Is Controlled by a SlMYB12-Regulated Transcriptional Network
Source: PLoS Genet. 2009 Dec 18;5(12):e1000777. doi: 10.1371/journal.pgen.1000777 (PMC2788616; doi:10.1371/journal.pgen.1000777)
Supplement: Figure S7 — Cuticular wax composition in the y mutant and wt fruit peel at three tested stages of fruit development, as analyzed by GC-MS/FID. Wax constituents are sorted by compound classes according to carbon number in the chains (n = 5, error bars indicate standard errors). (0.32 MB PPT) [file pgen.1000777.s007.ppt]

## Slide 1
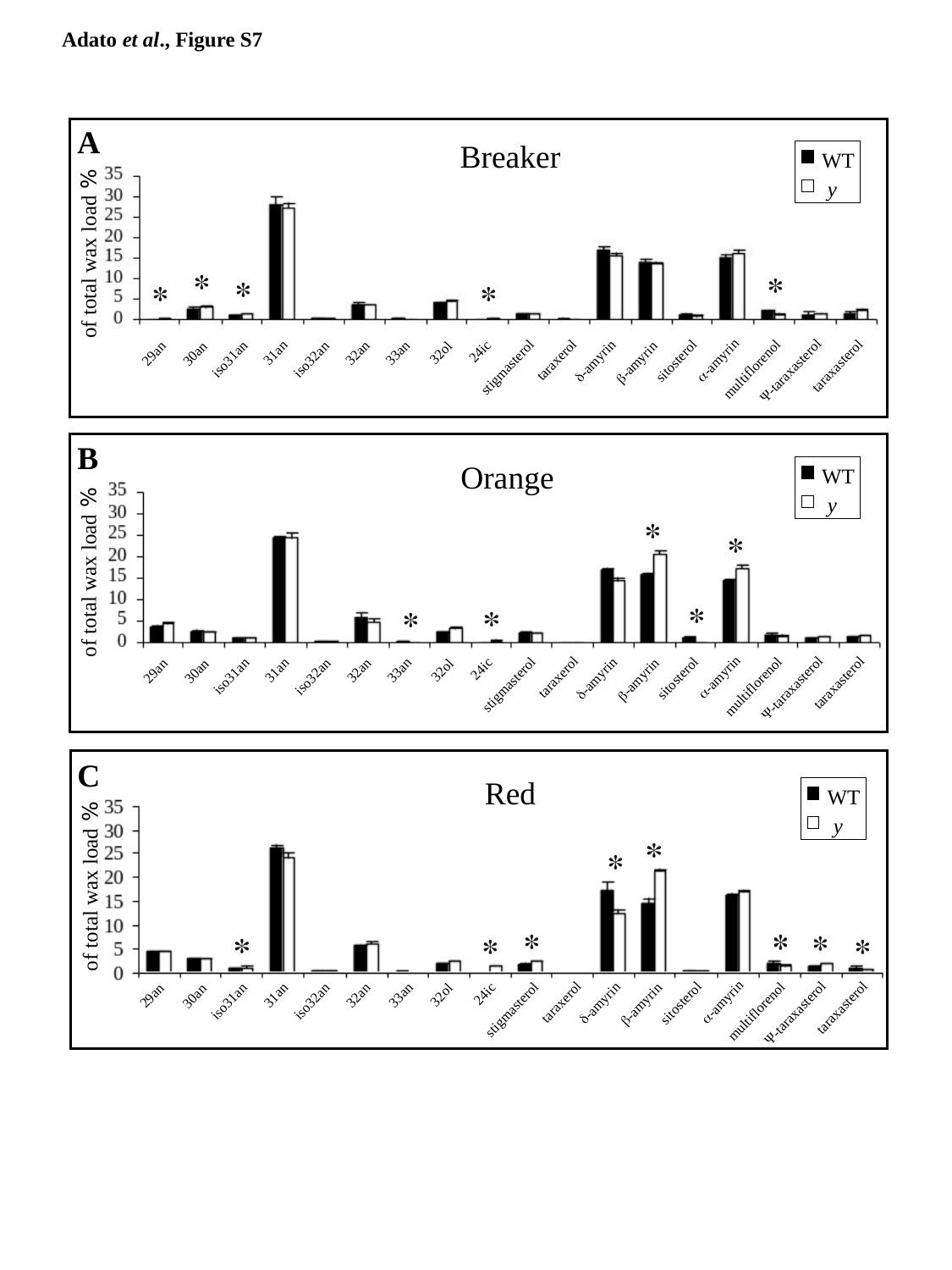

Adato et al., Figure S7
A
Breaker
WT
y
% of total wax load
24ic
32ol
31an
33an
32an
29an
30an
iso31an
iso32an
taraxerol
α-amyrin
-amyrin
sitosterol
β-amyrin
taraxasterol
stigmasterol
multiflorenol
Ψ-taraxasterol
B
Orange
WT
y
% of total wax load
24ic
32ol
31an
33an
32an
29an
30an
iso31an
iso32an
taraxerol
α-amyrin
-amyrin
sitosterol
β-amyrin
taraxasterol
stigmasterol
multiflorenol
Ψ-taraxasterol
C
Red
WT
y
% of total wax load
24ic
32ol
31an
33an
32an
29an
30an
iso31an
iso32an
taraxerol
α-amyrin
-amyrin
sitosterol
β-amyrin
taraxasterol
stigmasterol
multiflorenol
Ψ-taraxasterol
