# Supplementary material for: Fruit-Surface Flavonoid Accumulation in Tomato Is Controlled by a SlMYB12-Regulated Transcriptional Network
Source: PLoS Genet. 2009 Dec 18;5(12):e1000777. doi: 10.1371/journal.pgen.1000777 (PMC2788616; doi:10.1371/journal.pgen.1000777)
Supplement: Figure S8 — SlMYB12 assignment to tomato chromosome 1. (A) BstBI digestion of SlMYB12 genomic fragments amplified from the tomato set of interspecific introgression lines between cv. M82 and Lycopersicon pennellii [58]. (B) The Il1-1 pennellii chromosome segment, which does not overlap with other introgression lines resides between 17 CM to 41 CM. (0.31 MB PPT) [file pgen.1000777.s008.ppt]

## Slide 1
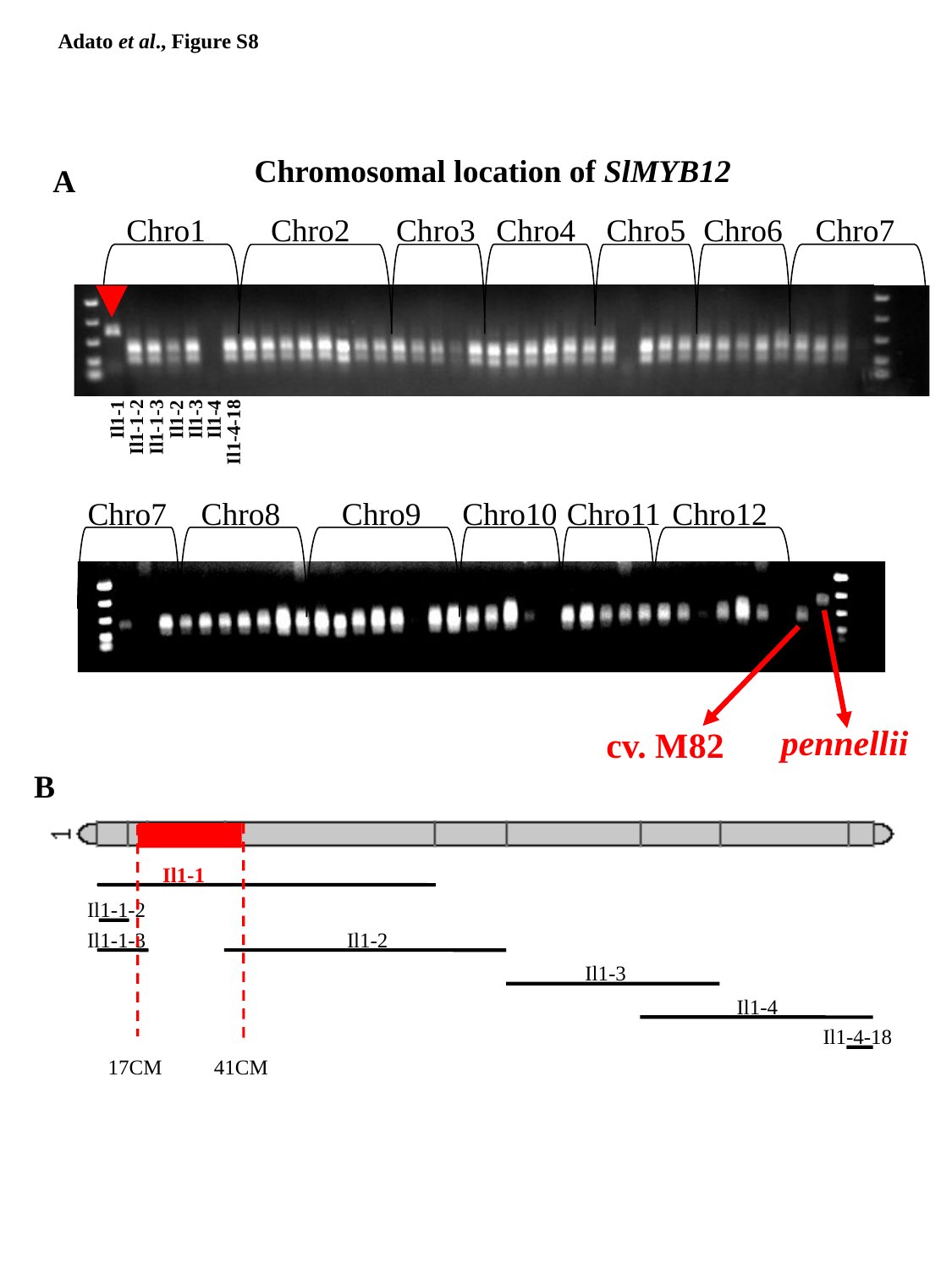

Adato et al., Figure S8
Chromosomal location of SlMYB12
Chro1
Chro2
Chro3
Chro4
Chro5
Chro6
Chro7
Il1-1
Il1-2
Il1-3
Il1-4
Il1-1-2
Il1-1-3
Il1-4-18
A
Chro7
Chro8
Chro9
Chro10
Chro11
Chro12
pennellii
cv. M82
B
Il1-1
Il1-1-2
Il1-1-3
Il1-2
Il1-3
Il1-4
Il1-4-18
17CM
41CM
