# Supplementary material for: Fruit-Surface Flavonoid Accumulation in Tomato Is Controlled by a SlMYB12-Regulated Transcriptional Network
Source: PLoS Genet. 2009 Dec 18;5(12):e1000777. doi: 10.1371/journal.pgen.1000777 (PMC2788616; doi:10.1371/journal.pgen.1000777)
Supplement: Figure S9 — Multiple alignment of flavonoid-related MYB transcription factors and the SlMBY12 alleles. (A) Multiple alignment of the flavonoid related factors that were down-regulated in the y mutant (tomato THM27/MYB4, MYB4-like and MYB12 putative protein products of TC174616 and other gene sequences that were reconstructed in this study), their closest paralogue (putative protein product of SlMYB12-like that was reconstructed in this study) and protein products of their Arabidopsis orthologues (MYB11 and MYB12, NP_191820 and NP_182268, respectively) as well as the predicted protein product of the SlMYB12 allele, y-1. Amino acid (aa) substitutions and the aa deletion in the y-1 allele are indicated in pink. (B) Nucleic acids alignment of the two SlMYB12 alleles. Exonic and intronic sequence differences are highlighted in grey. Red asterisks and underlines indicate premature polyadenylation (pad) sites of the y-1 transcripts. Out of 20 sequenced RACE products 4, 4, 3, 4, 3 were pad1a, pad2a, pad3a, pad4a, pad5a, pad6a versions and the short wt version, respectively. (0.09 MB PPT) [file pgen.1000777.s009.ppt]

## Slide 1
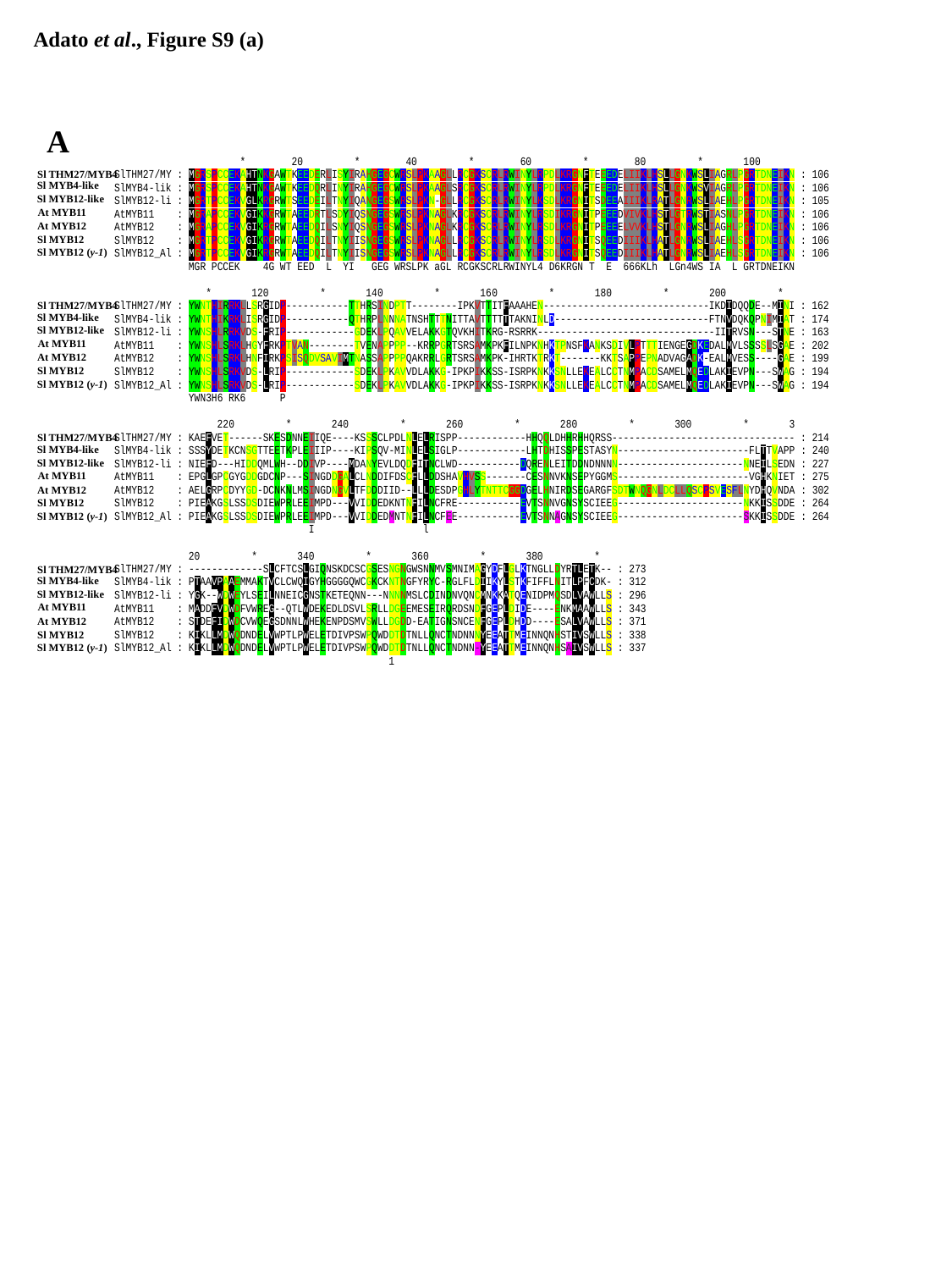

Adato et al., Figure S9 (a)
A
Sl THM27/MYB4
Sl MYB4-like
Sl MYB12-like
At MYB11
At MYB12
Sl MYB12
Sl MYB12 (y-1)
Sl THM27/MYB4
Sl MYB4-like
Sl MYB12-like
At MYB11
At MYB12
Sl MYB12
Sl MYB12 (y-1)
Sl THM27/MYB4
Sl MYB4-like
Sl MYB12-like
At MYB11
At MYB12
Sl MYB12
Sl MYB12 (y-1)
Sl THM27/MYB4
Sl MYB4-like
Sl MYB12-like
At MYB11
At MYB12
Sl MYB12
Sl MYB12 (y-1)

## Slide 2
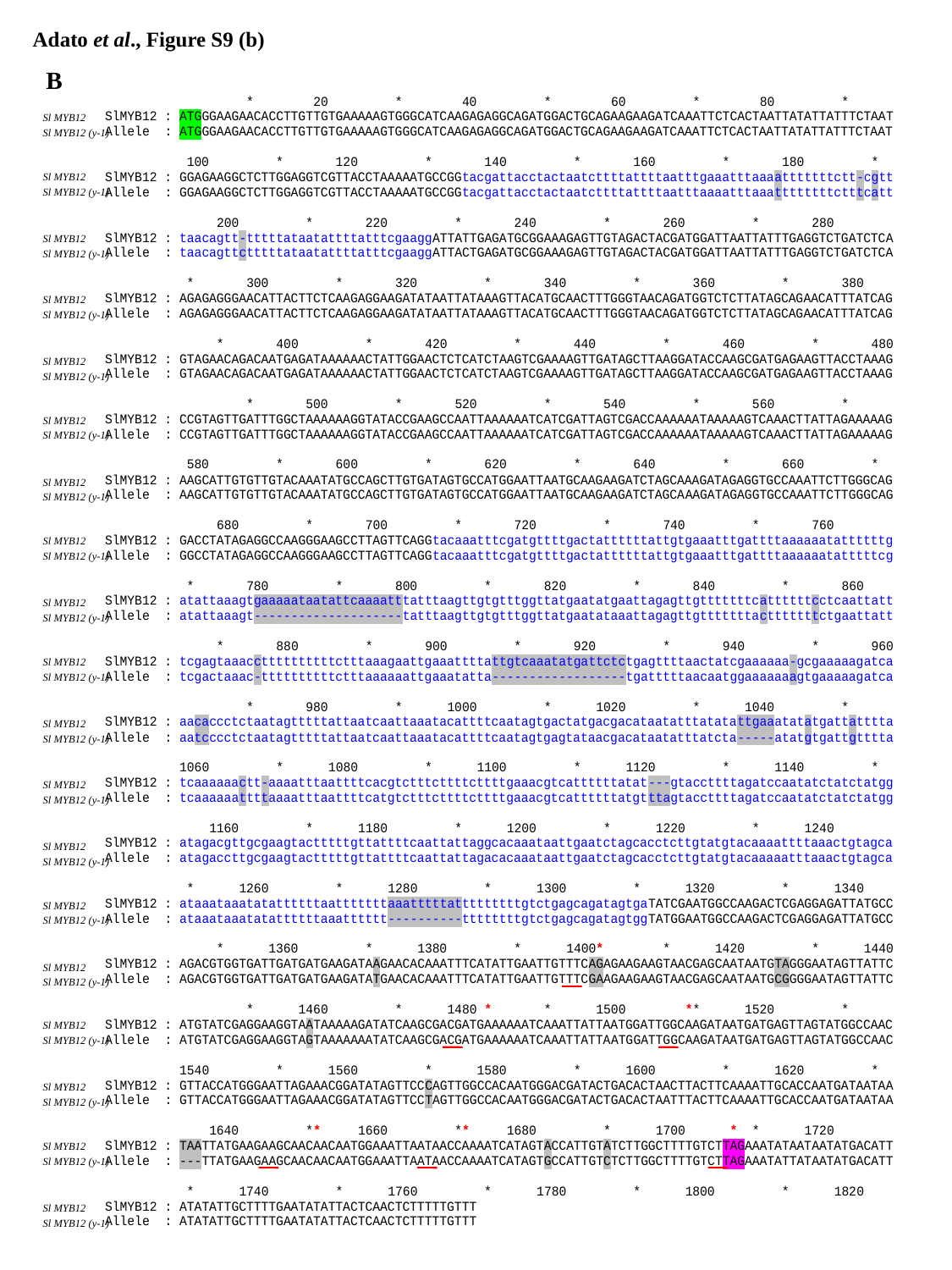

Adato et al., Figure S9 (b)
B
Sl MYB12
Sl MYB12 (y-1)
Sl MYB12
Sl MYB12 (y-1)
Sl MYB12
Sl MYB12 (y-1)
Sl MYB12
Sl MYB12 (y-1)
Sl MYB12
Sl MYB12 (y-1)
Sl MYB12
Sl MYB12 (y-1)
Sl MYB12
Sl MYB12 (y-1)
Sl MYB12
Sl MYB12 (y-1)
Sl MYB12
Sl MYB12 (y-1)
Sl MYB12
Sl MYB12 (y-1)
Sl MYB12
Sl MYB12 (y-1)
Sl MYB12
Sl MYB12 (y-1)
Sl MYB12
Sl MYB12 (y-1)
Sl MYB12
Sl MYB12 (y-1)
Sl MYB12
Sl MYB12 (y-1)
Sl MYB12
Sl MYB12 (y-1)
Sl MYB12
Sl MYB12 (y-1)
Sl MYB12
Sl MYB12 (y-1)
Sl MYB12
Sl MYB12 (y-1)
