# Supplementary material for: Fruit-Surface Flavonoid Accumulation in Tomato Is Controlled by a SlMYB12-Regulated Transcriptional Network
Source: PLoS Genet. 2009 Dec 18;5(12):e1000777. doi: 10.1371/journal.pgen.1000777 (PMC2788616; doi:10.1371/journal.pgen.1000777)
Supplement: Figure S10 — Total Ion Chromatograms of y, cv. AC, 35S:amiR-SlMYB12 and cv. MT peel samples at the red stage of fruit development, acquired in negative mode by the UPLC-QTOF-MS instrument. Putative identifications of the differential compounds are: 1- quercetin-dihexose-deoxyhexose, 2- quercetin-hexose-deoxyhexose-pentose, 3- quercetin rutinoside (rutin), 4- phloretin-di-C-hexose, 5- kaempferol-glucose-rhamnose, 6- naringenin chalcone, 7- dicaffeoylquinic acid III, 8- tricaffeoylquinic acid. Red and blue numbers indicate metabolites that showed elevated or reduced levels in the mutant/transgene samples compared to those of their corresponding wt. Black numbers indicate metabolites that did not differ between the y mutant and its corresponding cv. AC wt. (0.16 MB PPT) [file pgen.1000777.s010.ppt]

## Slide 1
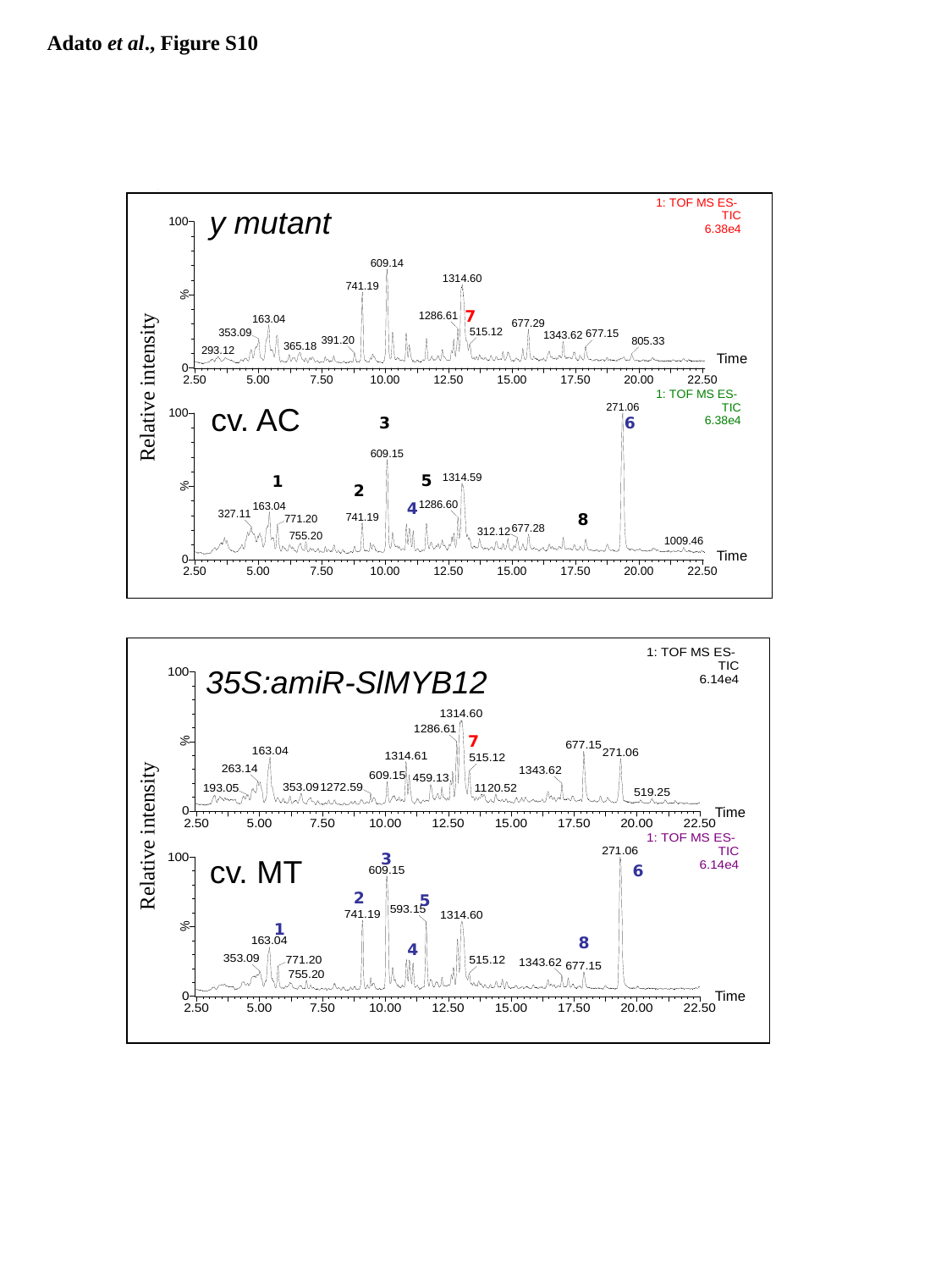

Adato et al., Figure S10
y mutant
7
Time
cv. AC
6
3
5
1
2
4
8
Time
Relative intensity
35S:amiR-SlMYB12
7
Time
Relative intensity
3
cv. MT
6
2
5
1
8
4
Time
