# Supplementary material for: Fruit-Surface Flavonoid Accumulation in Tomato Is Controlled by a SlMYB12-Regulated Transcriptional Network
Source: PLoS Genet. 2009 Dec 18;5(12):e1000777. doi: 10.1371/journal.pgen.1000777 (PMC2788616; doi:10.1371/journal.pgen.1000777)
Supplement: Figure S11 — RT-PCR relative transcript expression analyses of SlTHM27 (previously described by Mintz-Oron et al. [9], SlMYB4-like and SlMYB12-like in wt peel and flesh during five stages of fruit development. (0.06 MB PPT) [file pgen.1000777.s011.ppt]

## Slide 1
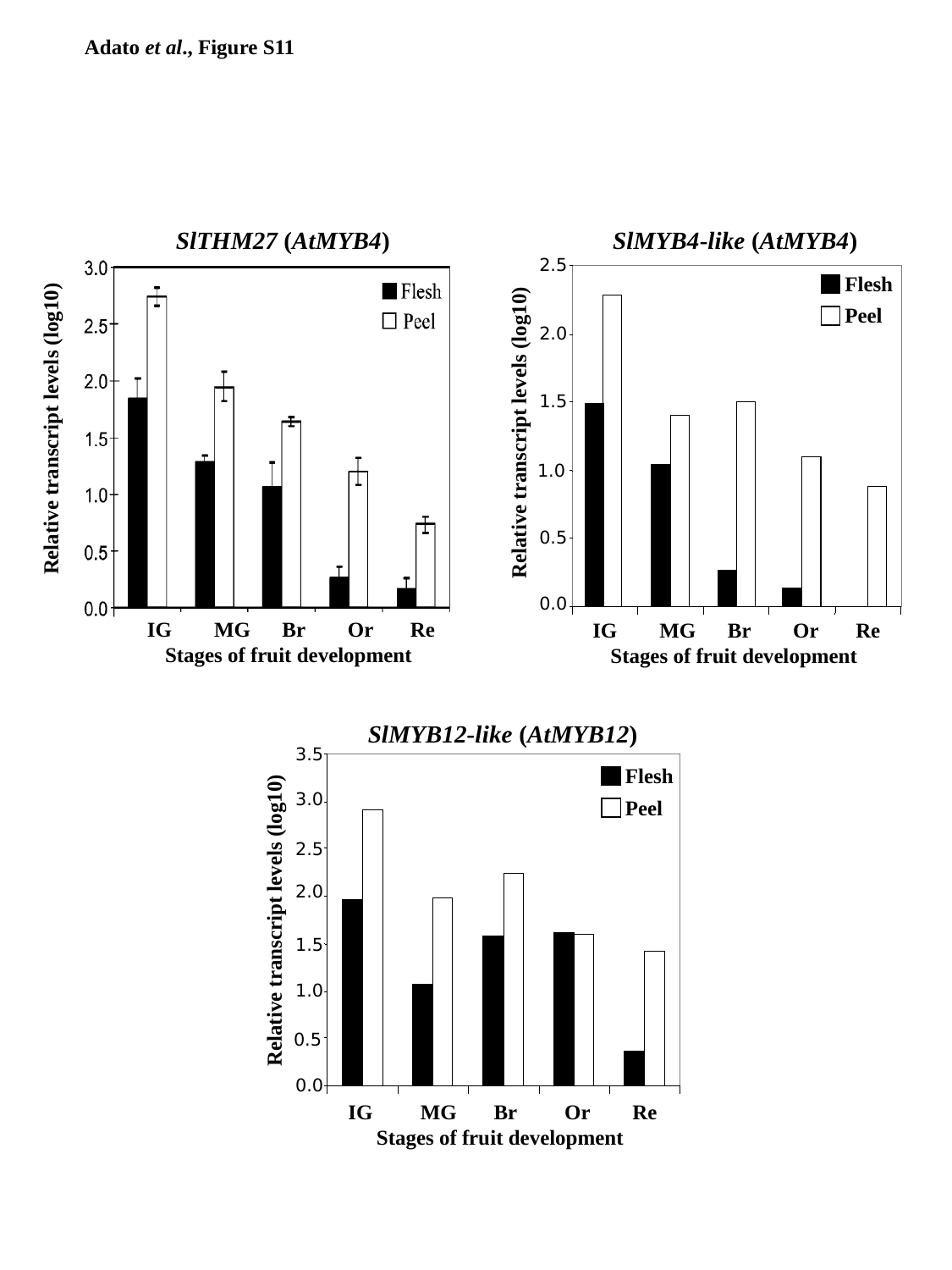

Adato et al., Figure S11
SlTHM27 (AtMYB4)
SlMYB4-like (AtMYB4)
2.5
Flesh
Peel
2.0
1.5
Relative transcript levels (log10)
Relative transcript levels (log10)
1.0
0.5
0.0
IG MG Br Or Re
Stages of fruit development
IG MG Br Or Re
Stages of fruit development
SlMYB12-like (AtMYB12)
3.5
Flesh
Peel
3.0
2.5
2.0
Relative transcript levels (log10)
1.5
1.0
0.5
0.0
IG MG Br Or Re
Stages of fruit development
