# Supplementary material for: Fruit-Surface Flavonoid Accumulation in Tomato Is Controlled by a SlMYB12-Regulated Transcriptional Network
Source: PLoS Genet. 2009 Dec 18;5(12):e1000777. doi: 10.1371/journal.pgen.1000777 (PMC2788616; doi:10.1371/journal.pgen.1000777)
Supplement: Table S3 — Summary of phenylpropanoid/flavonoid-related transcripts. (0.12 MB DOC) [file pgen.1000777.s014.doc]

| **Table S3. Summary of phenylpropanoid/flavonoid-related transcript and their expression detected by arrays and RT-PCR** | | | | | | | | |
| --- | --- | --- | --- | --- | --- | --- | --- | --- |
|  | **Gene** | | | **Microarray** | | **Real-Time PCR** | | **Real-Time**  **Primers** |
| **#** | **Short name- gene** | **Gene Annotation** | **TC** | **Peel** | **Flesh** | **Peel** | **Flesh** |  |
| 1 | CM | Chorismate Mutase | CK715539 | ↓ | ↔ |  |  |  |
| 2 | CM | Chorismate Mutase | TC174527 | ↔ | ↔ |  |  |  |
| 3 | PDH | Prephenate Dehydratase | TC172766 | ↓ | ↔ | ↓ | ↔ | F;GAGTACATCGCCGCCAACA (977)  R; AGTCACGTTGCTTGAATCATCCT (978) |
| 4 | PDH | Prephenate Dehydratase | TC180389 | ↔ | ↔ |  |  |  |
| 5 | PAR2 | Phenylacetaldehyde Reductase | BT013872.1 | ↑ | ↔ | ↔ | ↔ | F; CCCTGGATGGAGCTAAGGAGA (1845)  R; CCTTCACACCCCTCAACAACA (1846) |
| 6 | PAL | Phenylalanine Ammonia-Lyase | TC170429 | ↓ | ↔ | ↓ | ↔ | F; CAGCCTAAGGAAGGACTTGCA (965)  R; GAAAATCGCTGACAAGACTTCAGA (966) |
| 7 | PAL | Phenylalanine Ammonia-Lyase | TC172772 | ↓ | ↔ |  |  |  |
| 8 | C4H | Cinnamate 4-Hydroxylase | TC190665 |  |  | ↔ | ↔ | F; TCACGTCCACGTAACGTTGTG (689)  R; TGATACGTCTCATTTTTCTCCAATG (690) |
| 9 | C3H | P-Coumaroyl 3'-Hydroxylase | TC183733 | ↓ | ↔ | ↓ | ↔ | F; CACACTTTGGCTCGCAAACA (981)  R; CATATCCCATAGGAGGCCGATA (982) |
| 10 | COMT1 | Caffeic Acid 3-O-Methyltransferase | TC175188 |  |  | ↔ | ↔ | F; TTACCCTGGCGTTGAACACA (1518)  R; TGCTCATCGCTCCAATCATG (1519) |
| 11 | COMT2 | Caffeic Acid 3-O-Methyltransferase | TC177389 |  |  | ↔ | ↔ | F; TGACCTACCCAATGTCATCAAAGAT (1537)  R; GAATGATTAGCTCCCCTTGAGGA (1538) |
| 12 | 4CL | 4-Coumarate CoA Ligase | TC173193 | ↓ | ↔ | ↓ | ↓ | F; AACCCCACTGCTAAGGCTATTTT (691)  R; GACAATTACCCCCAAATGTCCTAA (692) |
| 13 | 4CL | 4-Coumarate CoA Ligase | TC173154 | ↔ | ↓ |  |  |  |
| 14 | 4CL | 4-Coumarate CoA Ligase | TC176157 | ↔ | ↔ |  |  |  |
| 15 | CHS1 | Chalcone Synthase | TC170658 | ↓ | ↓ | ↓ | ↓ | F; TGGTCACCGTGGAGGAGTATC (665)  R; GATCGTAGCTGGACCCTCTGC (666)  F*; GCATATCCACCATTTTTTCCGGC (410)  R*; CCCACAATGTAAGCCCAGCCC (402) |
| 16 | CCR | Cinnamoyl CoA Reductase-Like | TC180112 | ↓ | ↓ | ↓ | ↓ | F; CACGGAACGAATGGCATTTA (971)  R; TTCCAGATGCATGCAAGTAGAGA (972) |
| 17 | CCR | Cinnamoyl CoA Reductase-Like | TC178150 | ↓ | ↔ |  |  |  |
| 18 | CCR | Cinnamoyl CoA Reductase | TC170723 | ↓ | ↔ |  |  |  |
|  |  |  |  |  |  |  |  |  |
| 19 | REF1 | Reduced Epidermal Fluorescence | TC181878 | ↔ | ↔ | ↔ | ↔ | F; TTGGCGATCCCTTCAAGAAA (2155)  R; AGAGCTGTCACGGCCTTCTC (2516) |
| 20 | CHI | Chalcone Isomerase/ chalcone-flavanone isomerase | TC178705 | ↓ | ↓ | ↓ | ↓ | F; GTGCTTCTGGGAGTGCAAAGA (960)  R; CCCTTGTTCCACCTAAGTACCATT  (959) |
| 21 | CHI | Chalcone Isomerase | TC177570 | ↔ | ↔ |  |  |  |
| 22 | CHI | Chalcone Isomerase | NP840677 | ↔ | ↔ |  |  |  |
| 23 | F3H | Flavanone 3-Hydroxylase | TC180957 | ↓ | ↓ | ↓ | ↓ | F; CTGTTCAGCCCGTTGAAGGT (1070)  R; ACCACTGCTTGATGATCAGCAT (1071) |
| 24 | F3H | Flavanone 3-Hydroxylase | TC181836 | ↑ | ↔ |  |  |  |
| 25 | F3H | Flavanone 3-Hydroxylase | TC178533 | ↔ | ↔ |  |  |  |
| 26 | F3'H-like | Flavonoid 3'- Hydroxylase-Like | TC175149 |  |  | ↓ | ↓ | F; ATTCGCCGACGGTACTAACG (1129)  R; ATCGCCGATGTTGAAAACG (1130) |
| 27 | FLS | Flavonol Synthase | TC172800 | ↓ | ↔ | ↓ | ↓ | F; GAGCATGAAGTTGGGCCAAT (675)  R; TGGTGGGTTGGCCTCATTAA (676) |
| 28 | ANS-like | Anthocyanidin Synthase-Like | TC175220 |  |  | ↓ | ↓ | F; TTGGTTTGGAAGGCCATGAA (1127)  R; AAATCAGGCCTTGGACATGGT (1128) |
| 29 | 3GT | Flavonoid 3-Glucosyl Transferase | TC176277 | ↓ | ↓ | ↔ | ↔ | F; TCACAAGCCTACTTAATTTGTTCCA (961)  R; GCTCGAGGGAAAGTTCTAGATGAA (962) |
| 30 | 3GT | Flavonoid 3-Glucosyl Transferase | TC176549 | ↓ | ↔ | ↔ | ↔ | F; TGGGATGGCGTCAAACAAG (973)  R; CCCTGTTTCCTCCTCTGCTTCT (974) |
| 31 | RT | RhamnosylTransferase | TC179039 | ↓ | ↔ | ↓ | ↔ | F; TGCAGGATTCAGTTCAGTGATAGAG (969)  R; TCATATCCCCACTCACTAGTTTTGC (970) |
| 32 | Sl_JAF13 | Sl_JAF13 | TC182581 + TC190452 + TC185386 + TC178931 | ↔ | ↔ | ↔ | ↔ | F; GCAATCTTCTGGTCAACTGCAG  R; CCCGCCTGAACAGTCTTCC  F*; GAATATATGCCAAGTTGTAGCAAGTC  R*; CACAAAAAAGTGATGATCATGAAAG |
| 33 | Sl_MYB12-like | Sl_MYB12-like | AI771790 |  |  | ↔ | ↔ | F; CCAAACGAGGACGCAGTAGAA  R; ATGCCATAACATCTGGTCATCAAT |
| 34 | Sl_MYB12 | Sl_MYB12 | TC172990 |  |  | ↓ | ↔ | F; GCCAGCTTGTGATAGTGCCAT (1171)  R; AAGGCTTCCCTTGGCCTCTA (2174)  F*: ATGGGAAGAACACCTTGTTGT (1600)  R*: TCAAAAGCAATATATAATGTCATA (1975) |
| 35 | Sl_MYB4-like | Sl_MYB4-like | TC184379 | ↓ | ↔ | ↓ | ↔ | F; AGGGCTTCCCTTGGCTTCTA (889)  R; ATTGATGAGGCGTTGGTCTTCTT (890)  F*; CAAAGTATGGGACGTTCACC (1053)  R*; CCACCATGATATCCATTTGC (1054) |
|  |  |  |  |  |  |  |  |  |
| 36 | THM27 | THM27 | TC174616 | ↓ | ↔ | ↓ | ↔ | F; GTAAAGATTGCAGTTGTGGAAGTGA (887)  R; TTCAAGCCCAAAAAGTCATAACC (888)  F*; CCATTATCCTTCTCTCAATTGG (1051)  R*; CTATATTGCAAAGTTTACAACCATG (1052) |
| 37 | Sl_ANT2 | Sl_ANT2 | TC186580 |  |  | ↔ | ↔ | F;CCAGGAAGGACAGCAAACGA (1190)  R;CGAGGACGAGAATGAGGATGTAG (1191)  F*; GAATACTCCTATGTGTGCATC  R*; CAAAAATAAAAATTCTTTAATTAAGT |
| 38 | Sl_MYB111 | Sl_MYB111 | TC178481 |  |  | ↔ | ↔ | F; TCCTGATCTCAAACATGGGAAAAT (1173)  R; TTTTTCGGGCCATTCTTGAC (1174)  F*; GATGGTGCAAGAAGAAATAATGAG  R*; CGATAGCGAAAATATGTCACATTG |
| 39 | Sl_MYB61 | Sl_MYB61 | AW626100 + TC183887 |  |  | ↔ | ↔ | F; TGGCTGTTGGAGCTCTGTCC (1526)  R; CTCTTTTCAAATCAGGCCTCAAG (1527)  F*; GGCCGGGAGAGGCTTTTAT  R*; CTATCATATACCATCCACAAAAG |

↔ no difference between *y* and wild type

↑ up-regulated in the *y* mutant

↓ down-regulated in the *y* mutant

All RT-RCR expression analyses were carried out on cDNA from breaker fruit. Details regarding the developmental stages of altered transcript expression according to the micro-array can be found in table S1.
