# Supplementary material for: Fruit-Surface Flavonoid Accumulation in Tomato Is Controlled by a SlMYB12-Regulated Transcriptional Network
Source: PLoS Genet. 2009 Dec 18;5(12):e1000777. doi: 10.1371/journal.pgen.1000777 (PMC2788616; doi:10.1371/journal.pgen.1000777)
Supplement: Table S4 — Summary of isoprenoid-related transcripts. (0.05 MB DOC) [file pgen.1000777.s015.doc]

| **Table S4.** Summary of isoprenoid-related transcripts and their expression in peel and flesh of the *y* mutant and wild type fruit | | | | | | | | |
| --- | --- | --- | --- | --- | --- | --- | --- | --- |
|  | **Gene** | | | **Microarray** | | **Real-Time PCR** | | **Real-Time**  **Primers** |
| **#** | **Short gene name** | **Full name of gene** | **TC** | **Peel** | **Flesh** | **Peel** | **Flesh** |  |
| 1 | IPPI | Isopentenyl Diphosphate Isomerase | TC183769 | ↔ | ↑Re |  |  |  |
| 2 | IPPI | Isopentenyl Diphosphate Isomerase | TC188028 | ↔ | ↔ |  |  |  |
| 3 | IPPI | Isopentenyl Diphosphate Isomerase | TC175619 | ↔ | ↔ |  |  |  |
| 4 | PSY | Phytoene Synthase | TC183738 | ↔ | ↔ |  |  |  |
| 5 | PSY | Phytoene Synthase | TC178429 | ↔ | ↔ |  |  |  |
| 6 | PSY | Phytoene Synthase | TC171370 | ↔ | ↔ |  |  |  |
| 7 | PSY | Phytoene Synthase | TC181756 | ↔ | ↔ |  |  |  |
| 8 | ZDS | *ζ-*Carotene Desaturase | TC177671 | ↔ | ↔ |  |  |  |
| 9 | PDS | Phytoene Desaturase | TC171126 | ↔ | ↔ |  |  |  |
| 10 | LCY-B | Lycopene *β*-Cyclase | TC169910 | ↔ | ↔ |  |  |  |
| 11 | LCY-B | Lycopene *β*-Cyclase | TC173629 | ↔ | ↔ |  |  |  |
| 12 | LCY-ε | Lycopene *ε*-Cyclase | TC178153 | ↔ | ↔ |  |  |  |
| 13 | CRTR-B2 | *β*-Carotene Hydroxylase | TC170778 | ↓Br | ↓Br | ↓Br | ↔ | F; TTTCAGCCTCCGCTAGTTCC (1422)  R; CGGAGAGAAGAACAGAACCGG (1423) |
| 14 | CRTR-B1 | *β*-Carotene Hydroxylase | TC173604 | ↔ | ↔ |  |  |  |
| 15 | ZEP | Zeaxanthin Epoxidase | TC185210 | ↔ | ↔ |  |  |  |
| 16 | VDE | Violaxanthin Epoxydase | TC177173 | ↑Br | ↔ |  |  |  |
| 17 | NCED | 9-c*is*-Epoxycarotenoid Dioxygenase | TC169881 | ↓Br | ↓Re | ↓Br | ↔ | F; TGAACACCCTTTGCCGAAA (1420)  R; CGGTGACCGGAAGAGATTGA (1421) |

↔ no difference in expression between the y mutant and wild-type

↑ up-regulated in the *y* mutant

↓ down-regulated in the *y* mutant

The developmental stages at which genes expression was altered are indicated by Br and Re (breaker and red, respectively).
