# Supplementary material for: Fruit-Surface Flavonoid Accumulation in Tomato Is Controlled by a SlMYB12-Regulated Transcriptional Network
Source: PLoS Genet. 2009 Dec 18;5(12):e1000777. doi: 10.1371/journal.pgen.1000777 (PMC2788616; doi:10.1371/journal.pgen.1000777)
Supplement: Table S5 — Targeted HPLC-PDA isoprenoid analysis. (0.04 MB DOC) [file pgen.1000777.s016.doc]

| **Table S5. *Targeted HPLC isoprenoid analysis***  Retention time, spectral characteristic and the wavelength, used for the quantitation of the isoprenoids by HPLC. | | | | |
| --- | --- | --- | --- | --- |
| **Peak no.** | **Isoprenoid** | **RT (min)** | **Spectral Characterisation (nm at λmax)** | **λ (nm) used for quantification** |
| 1 | δ-Tocopherol^a^ | 7.81 | 299 | 296ex , 340em |
| 2 | γ-Tocopherol^a^ | 8.93 | 299 | 296ex , 340em |
| 3 | α-Tocopherol^a^ | 10.56 | 291.9 | 296ex , 340em |
| 4 | Chlorophyll *b*^a^ | 12.67 | 470.6 , 649.8 | 650 |
| 5 | Lutein^a^ | 13.47 | 443.9 , 473 | 445 |
| 6 | Chlorophyll *a*^a^ | 16.48 | 433 , 664.5 | 665 |
| 7 | Phytoene^b^ | 19.79 | 276 , 287.1 , 299 | 287 |
| 8 | Phytofluene^b^ | 21.06 | 332.4, 349.1 , 365.6 | 350 |
| 9 | ζ-Carotene^b^ | 26.04 | 380 , 402.9 , 425.8 | 401 |
| 10 | *trans*-β-Carotene^a^ | 26.57 | 452.4 , 479.1 | 452 |
| 11 | *trans*- lycopene^a^ | 37.53 | 447.5 , 473 , 504.6 | 473 |

a - Identified by comparison of the UV spectrum and retention time to the standard, injected under the same conditions

b - Putatively assigned by comparison to the spectral data, presented by Fraser et al.

(2000)
